# One-Class Genetic Algorithm for Authentication Analysis of Spectrochemical Data

**DOI:** 10.1021/acsomega.5c07696

**Published:** 2025-12-31

**Authors:** José R. de Morais Filho, Camilo de L. M. de Morais, Anne B. F. Câmara, Kássio M. G. Lima

**Affiliations:** † Biological Chemistry and Chemometrics, Institute of Chemistry, 28123 Federal University of Rio Grande do Norte, Natal, RN 5072-970, Brazil; ‡ Center for Education, Science and Technology of the Inhamuns Region, State University of Ceará, Tauá, CE 63660-000, Brazil

## Abstract

One-class classifier (OCC) models are widely applied to solve classification problems where control or class modeling from a target class is necessary. In this study, OCC models such as Data Driven Soft Independent Modeling of Class Analogy (DD-SIMCA) and One-Class Partial Least Squares (OCPLS) were associated with a new variable selection strategy, the one-class genetic algorithm (OGA), for classification analyses in three clinical applications: COVID-19, endometriosis, and dengue samples. DD-SIMCA was implemented in a rigorous approach, using α = 0.05, while OCPLS was performed with partial robust M-regression (PRM). For the three cases, a better classification performance was obtained using the OGA associated with the OCC model. The performance of the OGA-PRM-OCPLS showed better results for both COVID-19 and endometriosis cases when compared to DD-SIMCA, with a classification sensitivity of 100%. However, the best results for dengue classification were obtained by using the OGA-DD-SIMCA model (sensitivity = 100%). The selected variables obtained by the OGA can be used to relate this information to biomarkers capable of distinguishing between case and control groups. These findings have the potential to improve some disease diagnosis using chemometrics for the development of rapid, low-cost, and minimally invasive screening methodologies.

## Graphical abstract

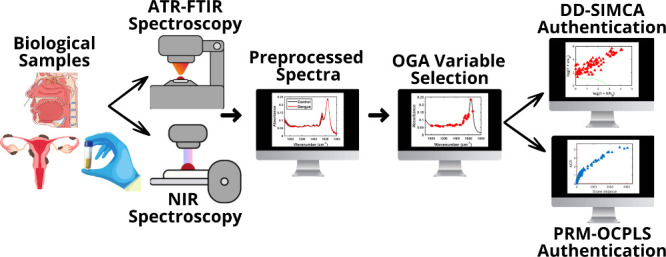

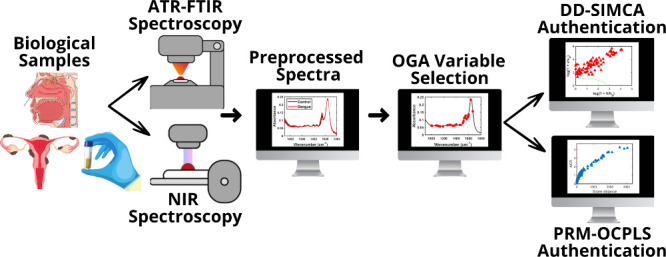

## 1 Introduction

Multivariate classification models are commonly employed across many scientific fields, such as food science, medical diagnostics, forensic analysis, microbiological studies, and fuel research, among others.^1–5^ In analytical chemistry, the aim of classification is to build categories, i.e., classes based on data acquired to characterize samples through a set of variables.

The term “class” denotes a group of samples that share similar features.^6^ Classification models are an important group of pattern recognition tools often applied in two-class or multiclass classifiers. Historically, they were the first multivariate models introduced and applied to qualitative analyses in pattern recognition. Their use has gained strength as relevant software has become more commercially available, seemingly providing better results compared with other models.^7^ Models such as Linear Discriminant Analysis (LDA), Quadratic Discriminant Analysis (QDA), Partial Least Squares Discriminant Analysis (PLS-DA), k-Nearest Neighbors (k-NN), and Support Vector Machines (SVM), among others, are known discriminant models.^7^

Class modeling (CM) or one-class classifier (OCC) models are an important set of models that answer the following general question: “can object O, declared of class A, really belong to class A?”^8^ For this, it is assumed that the term “class” denotes a set of samples that share similar features or attributes.^6^ Therefore, the class model is constructed using samples that undoubtedly belong to the target class, characterized by chemical, physical, and other relevant variables.^9^

Sensitivity and specificity are parameters that characterize a class model. Sensitivity measures the percentage of correct positive decisions (i.e., the ability to correctly identified true positives, thereby minimizing type I errors), while specificity measures the complementary percentage of correct negative decisions, thereby reducing type II errors.^9^ Models such as Soft Independent Modeling of Class Analogy (SIMCA) and two of its variants, Alternative SIMCA (Alt-SIMCA) and Data Driven SIMCA (DD-SIMCA), as well as Unequal Class Spaces (UNEQ), Potential Functions (PF), One-Class Support Vector Machines (OC-SVM), and One-Class Partial Least Squares (OCPLS), among others, are some examples of class modeling models.^6^

Discriminant analysis is composed of binary (conventional) and multiclass discrimination.^10^ The main distinction between discriminant binary and class modeling is that discriminant binary requires at least two classes to define the optimal boundary separating objects belonging to different classes. In contrast, class modeling can be approached in asymmetric cases, where a single class is represented in the training set, or in scenarios where only one class needs to be modeled.^6^ Furthermore, an enclosed class space is defined according to predetermined confidence levels, enabling the verification of compliance.^7^ When an object is tested against multiple modeled classes, it may be assigned to more than one class.^6^ To improve the classification models, variable selection algorithms can be applied to the data sets.

Variable selection algorithms are useful tools to find specific spectral markers associated with class separation. These algorithms enable the extraction of features from dominant spectral bands, effectively reducing the high redundancy and strong collinearity. By selecting representative variables, they allow grouping and replacement of the original set of variables, leading to a more concise, simple, and informative model, along with reducing the noise caused by irrelevant variables.^11,12^

Various types of variable selection strategies are employed in data sets, typically classified into three main groups: filter approaches, wrapper approaches, and embedded approaches. Filter approaches evaluate variable performance based on inherent properties prior to model building, generally by defining a ranking criterion and applying a threshold. Some examples include Variable Importance in Projection (VIP) and Selectivity Ratio (SR). Wrapper approaches, on the other hand, perform feature selection iteratively, as seen in strategies such as the Genetic Algorithm (GA). Lastly, embedded approaches integrate variable selection within the model development process, including models like Principal Component Analysis (PCA) and Partial Least Squares (PLS) regression.^11,13^

Herein, a new variable selection approach for class modeling is reported. This approach is a modification of the GA for discriminant analysis applied to a single class. This can be a useful approach to enhance class discrimination and spectral interpretability of complex data sets for authenticity applications or for modeling specific target classes.

## 2 Materials and Methods

### 2.1 DD-SIMCA

The Data Driven Soft Independent Modeling of Class Analogy (DD-SIMCA) is a variant of SIMCA, one of the most widely used one-class classification models in chemometrics.^6,14,15^ A comprehensive description of the DD-SIMCA theory can be found elsewhere.^15,16^

DD-SIMCA is based on the decomposition of preprocessed data.^16^ In this approach, PCA is applied to model the target class using the training data matrix **X** (*I* × *J*), as described in eq 1:

1 $$X=TP^{T}+E$$

where **X** is the preprocessed spectral data matrix of size (*I* × *J*), where *I* is the number of objects and *J* the number of variables; **T** is the scores matrix of size (*I* × *A*), where A is the number of principal components; **P** is the loadings matrix of size (*J* × *A*); and **E** (*I* × *J*) represents the residuals matrix. The superscript T denotes the matrix transpose.

Based on the PCA results, the score distance (SD), *h*_*i*_, and the orthogonal distance (OD), ν_*i*_, are calculated. The SD is defined as the squared Mahalanobis distance and reflects how far the projection of sample *i* lies from the origin of the principal component (PC) space. Although the Mahalanobis distance typically accounts for pairwise covariance between variables, this is not an issue in this context, as PCA scores are orthogonal by design.

The OD is defined as the squared Euclidean distance between sample *i* and the score subspace; that is, it represents how far the original data point is from its corresponding projection in the PC space.

The SD (*h*_*i*_) and OD (*v*_*i*_) can be calculated using eqs 2 and 3, respectively:

2 $$h_{i}=\overset{A}{\underset{a=1}{\sum }}\frac{t_{ia}^{2}}{\lambda_{a}}$$

3 $$v_{i}=\overset{J}{\underset{j=1}{\sum }}e_{ij}^{2}$$

where λ_*a*_ = **t**_*a*_^T^ **t**_*a*_ is the eigenvalue corresponding to component *a*, representing the sum of the squared score values for that component (*t*_*a*_); and *e* is the residual. In possession of the distances, the distance plot can be plotted as *h*/*h*_0_ vs *v*/*v*_0_ or log *h*/*h*_0_ vs log *v*/*v*_0_. The total distance is then calculated by eq 4:

4 $$c=N_{h}\frac{h}{h_{0}}+N_{v}\frac{v}{v_{0}}\propto X^{2}\left(N_{h}+N_{v}\right)$$

where parameters *v*_0_ and *h*_0_ are the scaling factors, and *N*_*h*_ and *N*_*v*_ represent the respective degrees of freedom (DoF). These parameters are unknown *a priori* and can be estimated using a data driven approach.^15,16^

The acceptance area (or decision threshold) for the target class is defined based on a predefined type I error rate, α. The acceptance condition is given by

5 $$c\leq c_{\mathrm{crit}}\left(\alpha \right)$$

where

6 $$c_{\mathrm{crit}}=X^{2}\left(1-\alpha ,N_{h}+N_{v}\right)$$

*X* ^2^ is the (1 – α) quartile of the chi-squared distribution with *N*_*h*_ + *N*_*v*_ degrees of freedom.^15,16^ After this step, the model is finalized and is ready for the classification of new data samples. It can be represented by an acceptance area in the orthogonal vs score distance space, also known as the acceptance plot, defined by the given α value. The α value specifies the type I error. Each sample in the training set can be characterized as regular, extreme, or outlier, depending on its location in the acceptance plot. A regular sample is one that is well described by the model and assigned to the target class. An extreme sample lies at the boundary of the acceptance area and may still belong to the target class but with higher variability. An alien sample, considered an outlier, is not attributed to the target class and is assumed to belong to an alternative meta-class.^15,16^ The second cutoff level is defined as the outlier boundary, constructed based on a specified γ value. This value represents the probability that at least one regular object from the training set will be erroneously classified as an outlier. Unlike the acceptance area, the outlier boundary depends on the size of the training set. The value for γ is 0.01.

The extreme plot and sensitivity plot can help assess the quality of classification models and support the selection of the optimal number of PCs.^15,16^

The classification results are shown in the acceptance plot. In addition, the value of the type II error (β), which represents the rate of incorrect acceptance of alien samples as target class objects, is calculated. A reverse evaluation is also possible, in which a specific type I error (α) corresponds to a given value of the type II error (β).^15^

### 2.2 OCPLS

The One-Class Partial Least Squares (OCPLS) classifier is a class modeling (CM) approach based on Partial Least Squares (PLS), offering performance comparable to that of SIMCA.^17,18^ However, data analysis may sometimes involve outliers and nonlinear contaminated data sets, which can lead to failures and bias in parameter estimation.^19^ To address these issues, variants such as the ordinary OCPLS, the nonlinear Gaussian Radial Basis Function (RBF or GRBF)-OCPLS, and the robust OCPLS based on partial robust M-regression (PRM) have been proposed in the literature.^17,19^

The OCPLS model is constructed as a special case of PLS regression, assuming that **X** (*m* × *n*) contains *m* objects described by *n* characteristic variables from the target class to be modeled:

7 $$1=Xb_{\mathrm{PLS}}+e$$

where **1** is the response vector (*m* × 1) with all elements equal to one, **b**_PLS_ (*n* × 1) contains the PLS regression coefficients, and **e** (*m* x 1) is the residual vector. The variables or features in **X** must not be centered; otherwise, all variables could become orthogonal to the constant response vector **1**.

**b**_PLS_ (*n* × 1) is computed by regression of **1** on *K* primary latent variables (LVs) or components as

8 T=XW

9 1=Tq

10 $$b_{\mathrm{PLS}}=\mathrm{Wq}$$

where the columns of **T** (*m* × *K*) contain the scores of *K* significant orthogonal LVs, **W** (*n* × *K*) holds the PLS loadings of the *K* LVs, and **q** (*K* × 1) denotes the regression coefficients relating **T** to the response vector **1**. The prediction of the residual sum of squares (PRESS) is obtained by Traditional Cross-Validation (TCV) to estimate the optimum number of LVs.

Distance measures are derived from the OCPLS model, two of which are the score distance (SD), which represents the position of an object in the space spanned by the primary OCPLS components, and the absolute centered residual (ACR), which serves as a measure of the dispersion of the projection onto the OCPLS regression coefficient vector. Based on these metrics, the upper control limit (UCL) is calculated for the ACR and used to plot and identify samples belonging to an alien class. The critic points of standard normal distribution with α = 0.05 and F-distribution were used to calculate the number of degrees of freedom.

The Gaussian Radial Basis Function (GRBF) is commonly used to develop a nonlinear OCPLS model. A transformation is applied and centered at the positions of training objects such that the number of GRBF equals the number of training samples. All variables must be rescaled to a range between 0 and 1 to implement the nonlinear GRBF-OCPLS model. The number of LVs and the kernel width parameter can be estimated simultaneously by analyzing the predicted residuals obtained through cross-validation. This cross-validation can be performed using a TCV approach or Monte Carlo Cross-Validation (MCCV).^19,20^

Partial robust M-regression (PRM) is an effective and reliable robust PLS model that downweights both orthogonal outliers and leverage objects. This leads to more accurate regression coefficients due to improved confidence levels, resulting in better model precision, especially when dealing with noisy data. In one-class classifiers, differences among regular objects are expected and must be allowed in order to capture the natural variation within the same class. Based on a predefined cutoff for the percentage of outliers, PRM is applied using all the training data. Consequently, the objects assigned the lowest weights by PRM are identified as outliers.^19–22^

The values of SD and ACR indicate whether an object lies inside or outside the modeled class region. According to the values of ACR and SD, the object in question can be assigned to one of four groups: regular or normal objects (region 1), which exhibit both a small SD and a small ACR; good leverage objects (region 2), which have a large SD and a small ACR; response outliers (region 3), characterized by a small SD and a large ACR; and bad leverage objects (region 4), which present a large SD and a large ACR. The ACR quantifies the distance of a sample from the OCPLS class model. Consequently, samples characterized by a low SD and a high ACR are classified as class outliers. Multivariate Statistical Quality Control (MSQC) can be useful for detecting objects falling in regions 2, 3, and 4 as different types of outliers.^17,19^

### 2.3 One-Class Genetic Algorithm (OGA)

The genetic algorithm (GA) is an iterative combinational algorithm inspired by Mendelian genetics, where a set of initial random variables (chromosomes) undergo processes like selection, crossover combinations, and mutations until the fittest set of variables is selected according to the minimization of a cost function.^23,24^ This cost function may vary; for example, it could be the root mean squared error of prediction (RMSEP) in regression models^25^ or the misclassification error for a given classifier in classification models.^26^ The aforementioned processes are repeated several times during generations until a specific set of initial variables that provide the best validation results are finally selected. Additionally, the nondeterministic nature of the GA may result in local minima, which could be avoided by running the algorithm several times in search of the best fitness value. Fitness herein is defined as the inverse of the cost function.

In the one-class genetic algorithm (OGA), a modification of the validation samples used to find the minimum cost is made so that the model is trained with a single class. For this, two pseudo-classes are created in the validation process: positive and negative. The positive class comprises the samples with smaller distances between them and the class center of the target class in a Euclidian space and, thus, the samples with profiles closer to the class mean. The negative class comprises samples with larger distances to the class center, such as outliers or borderline samples present in the target class. To make this selection during the GA process, the Kennard–Stone (KS) algorithm^27^ is applied to separate the positive and negative classes; the positive being the samples closer together and closer to the class mean, and the negative being the outer samples. After class separation, the cost function used to distinguish the classes was based on an LDA classifier defined as

11 $$G=\frac{1}{N_{v}}\overset{N}{\underset{n=1}{\sum }}g_{n}$$

where *N*_v_ is the number of validation samples and *g*_*n*_ is defined as

12 $$g_{n}=\frac{r^{2}\left(x_{n},m_{I\left(n\right)}\right)}{\min_{I\left(m\right)\neq I\left(n\right)}r^{2}\left(x_{n},m_{I\left(m\right)}\right)}$$

where *r* ^2^(*x*_*n*_, *x*_*I*(*n*)_) is defined as the squared Mahalanobis distance between sample *x*_*n*_ and the center of the positive class *m*_*I*(*n*)_, and *r* ^2^(*x*_*n*_, *x*_*I*(*m*)_) is the squared Mahalanobis distance between sample *x*_*n*_ and the center of the negative class *m*_*I*(*m*)_. GA was performed three times starting from different random initial populations, and the variables with the best fitness value were selected to build the further OCC models. The GA model was built using 100 generations with 200 chromosomes each. Crossover and mutation probabilities were set to 60% and 10%, respectively. The positive class was defined as the 70% of samples closer to the class center of the target class, while the negative class was defined as the remaining 30% of samples. The OGA schematic workflow is shown in Figure 1, where the steps until the selected variables are achieved is described.

**Figure 1:**
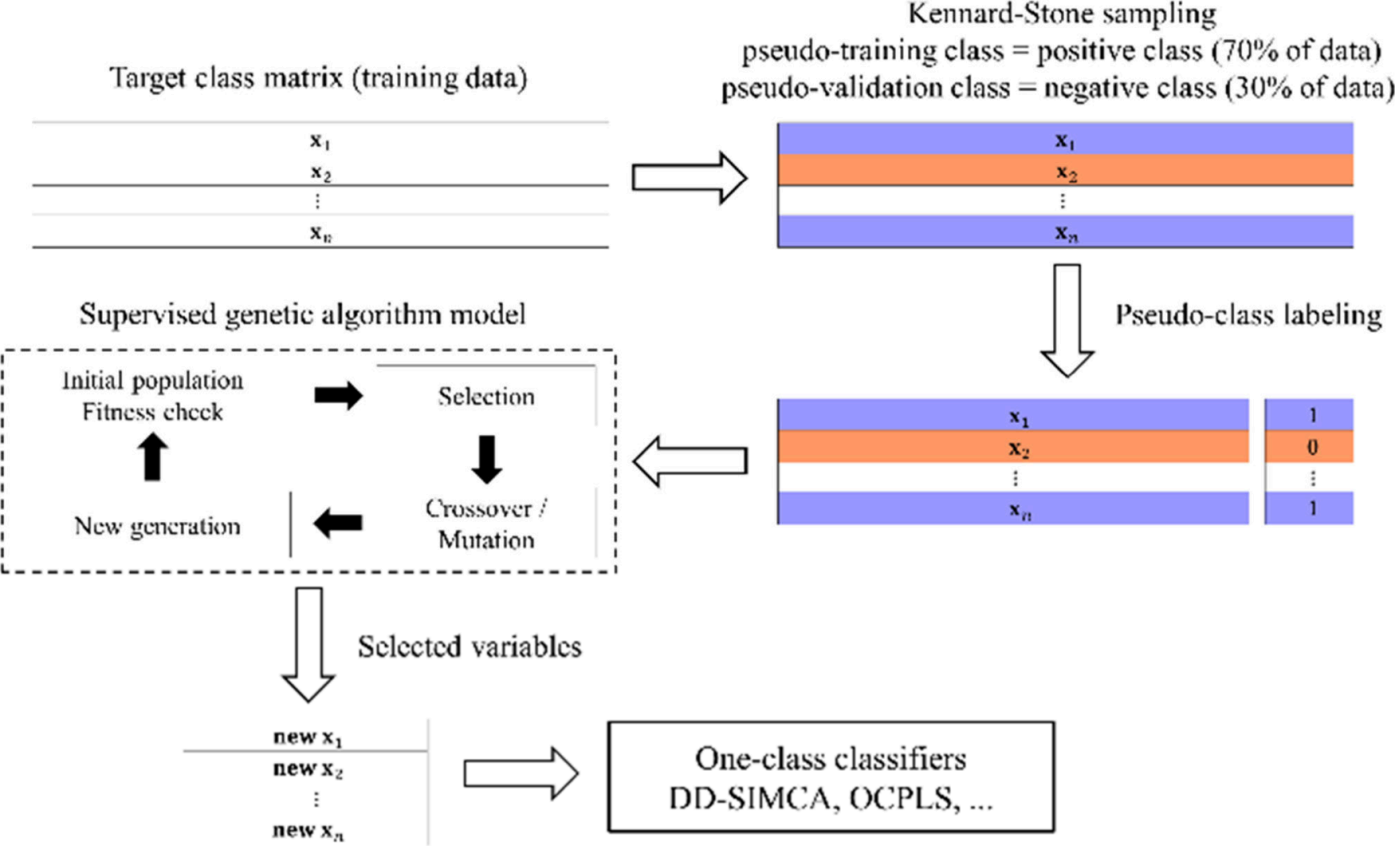
One-class genetic algorithm (OGA) schematic workflow.

### 2.4 Model Quality Evaluation

The performances of the DD-SIMCA and OCPLS models were evaluated under identical spectral preprocessing conditions, in terms of sensitivity (SEN) of an external prediction set, defined as

13 $$\mathrm{SEN}=\frac{\mathrm{number of true positives}}{\mathrm{number of target samples}}$$

where SEN is the ratio between the number of true positives and the number of target samples.^28,29^

All the models were built within the MATLAB R2024b environment (MathWorks, Inc., USA) using lab-made routines. Additionally, the DD-SIMCA and OCPLS models were built using the DD-SIMCA Toolbox^15^ and OCPLS Toolbox.^19^

### 2.5 Data Sets

#### 2.5.1 COVID-19 Data

The COVID-19 samples were made available by the Central Laboratory of Dr. Almino Afonso (LACEN), affiliated with the Federal University of Rio Grande do Norte (UFRN), Brazil. The samples were collected in public health units in the State of Rio Grande do Norte, Brazil, and the patients/volunteers read and signed the Free and Informed Consent Form. The study received ethical approval from the ethics committee under protocol number 65128122.​3.0000.​5537, and all procedures were conducted in conformity with the Declaration of Helsinki. To carry out the experiments, nasopharyngeal secretion was collected assisted by a nasal swab, fully sterilized, and carefully introduced into the nostril following rotation movements. Before the procedures, the swab was added to a salt solution, which was part of the collection kit. These samples were stored at a temperature range between 2 and 8 °C for a maximum of 3 days and stored at −80 °C in a freezer. This control-case study resulted in a total of 173 samples, with 84 for the healthy control group and 89 with a clinical diagnosis of COVID-19. All patients were confirmed as a control or case using the Reverse Transcription Polymerase Chain Reaction (RT-PCR).

NIR spectra were obtained using an ARCoptix FT-NIR Rocker spectrophotometer (Arcoptix S.A., Switzerland). The tests were carried out in transflectance mode, with a spectral resolution of 8 nm. The analytical procedure consisted of transferring 10 μL of the sample to an aluminum paper surface and using an optical fiber positioned onto each paper. The measurements were repeated 5 times. Then, the spectral data were cut in the 1000–2500 nm fingerprint and preprocessed with Extended Multiplicative Signal Correction (EMSC, first order polynomial fitting) and Savitzky–Golay smoothing (SG, window of 7 points with first order polynomial fitting was applied), with all parameters fixed.

#### 2.5.2 Endometriosis Data

The endometriosis samples were obtained at Januário Cicco Maternity School (MEJC), affiliated with the Federal University of Rio Grande do Norte (UFRN), Brazil. The study received ethical approval from the ethical committee at MEJC/UFRN under protocol no. 44352921.​6.0000.​5292, and all procedures were conducted in compliance with the Declaration of Helsinki. To conduct the experiments, venous blood samples were collected from each patient and then centrifuged at 3600 rpm for 7 min to separate the plasma. 100 μL aliquots of plasma were transferred to Eppendorf tubes and stored at −80 °C until the spectroscopic analysis. This control-case study resulted in a total of 75 samples, with 41 samples derived from women with a clinical diagnosis of endometriosis (case, *n* = 41) and 34 samples from the healthy control group (*n* = 34). The analytical procedure for acquiring the NIR spectra of endometriosis was developed as described in ref 30. The spectral data were preprocessed with MSC and SG smoothing (in MSC, the average spectrum was used, and in SG, a window of 15 points with first order polynomial fitting was applied), with all parameters fixed.

#### 2.5.3 Dengue Data

Dengue (*n* = 88) vs healthy control (*n* = 90) patients were also investigated using attenuated total reflection Fourier transform infrared (ATR-FTIR) spectra collected from blood samples. This data set is derived from Santos et al.^31^ and public available on the Figshare repository,^32^ whose spectroscopic data acquisition is available elsewhere.^31^ The spectroscopic data at the fingerprint region (900–1800 cm^–1^)^33^ were first analyzed to find possible outliers using the Hotelling’s *T* ^2^ vs *Q* residuals test.^23^ Two outliers were identified in the healthy controls data and removed; thus, the final data set contained 88 dengue and 88 healthy control samples. The spectral data were then preprocessed by MSC (the average spectrum was used) and SG smoothing (window of 7 points with first order polynomial fitting was applied), with all parameters fixed.

## 3 Results and Discussion

### 3.1 IR (NIR and ATR-FTIR) Spectroscopy

The preprocessed infrared spectra (MIR/NIR) for the three data sets applied in this study are depicted in Figure 2. The NIR spectra of the COVID-19 samples (Figure 2a) show the −CONH_2_ primary amides first and second overtones at 1450 and 2016 nm, respectively. The main difference between the two classes occurs due to the band displacement at 2363 nm, referring to the CH stretching and C–C stretching from lipids.^34^

**Figure 2:**
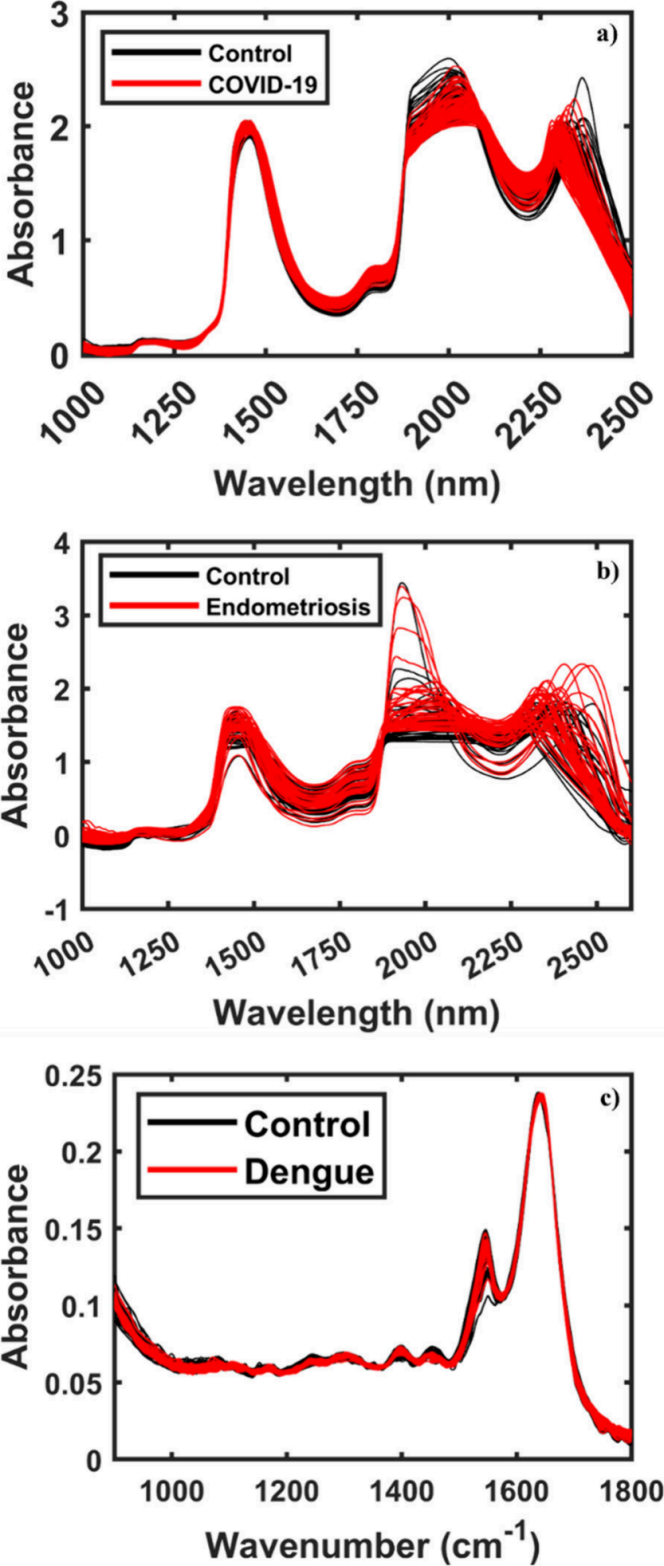
Preprocessed NIR spectra for (a) COVID-19, (b) endometriosis, and (c) dengue data sets.

In addition, for the NIR spectra of the endometriosis data set (Figure 2b), it is possible to observe the −CH_2_ second overtone at 1179 nm, as well as the first overtone of N–H stretching and the first overtone of O–H stretching. The feature at 1788 nm is associated with lipid structures. The band at 1950 nm corresponds to the second overtone of C–O stretching in carbohydrates, while the band at 2332 nm is related to the stretching and bending of CH associated with methylene.^30,34,35^

Furthermore, the ATR-FTIR spectra for the virus data set show four main bands. The first one, at 1641 cm^–1^, can be associated with amide I, while the second at 1547 cm^–1^ is related to the amide II band of proteins. In addition, the bands at 1396 and 1454 cm^–1^ correspond to the symmetric CH_3_ bending of methyl groups of proteins and asymmetric methyl deformation, respectively.^36,37^ However, for the three examples, it is possible to notice a high similarity between both spectra (case and control), indicating a spectral overlap from the groups when plotted together; thus, it is not possible to distinguish between them visually. For this reason, it is necessary to use a strategy capable of differentiating between the samples. In this context, the one-class classifier models are a powerful tool to identify samples of clinical interest (case group), since these models help to detach the target sample from all the other classes,^38^ especially when combined with variable selection methods, such as the OGA, developed in this study.

### 3.2 One-Class Models

The one-class classifier models were developed using Linear OCPLS, GRBF-OCPLS, PRM-OCPLS, and DD-SIMCA. The performance of these models for the COVID-19, endometriosis, and dengue data sets, both before and after variable selection using OGA, was evaluated using a rigorous approach.^29^ This strategy was defined with a significance level of α = 0.05, and the results for the quality parameters are presented in Table 1.^29^ The SEN value denotes the percentage of target class samples that are correctly classified as belonging to the target class.^20^

**Table 1:** Model Quality Parameter Results for the COVID-19, Endometriosis, and Dengue Data Sets Using Four One-Class Modeling Approaches: Linear OCPLS, GRBF-OCPLS, PRM-OCPLS, and DD-SIMCA, Applied Both before and after Variable Selection Using OGA

	OCPLS						DD-SIMCA	
	Linear	Linear-OGA	GRBF	GRBF-OGA	PRM	PRM-OGA	Solo	OGA
COVID-19								
SEN TRAIN^a^ (%)	96.82	93.65	100.00	93.65	98.28	94.83	91.80	93.55
SEN PRED.^b^ (%)	100.00	96.15	100.00	100.00	100.00	88.46	100.00	100.00
Endometriosis								
SEN TRAIN (%)	93.10	93.10	100.00	96.55	92.86	92.59	97.06	91.18
SEN PRED. (%)	100.00	100.00	100.00	100.00	100.00	100.00	91.67	100.00
Dengue								
SEN TRAIN (%)	95.08	93.44	100.00	96.72	100.00	98.15	93.22	94.92
SEN PRED. (%)	100.00	100.00	100.00	100.00	100.00	100.00	96.30	92.60

a SEN TRAIN, sensitivity of training.

b SEN PRED., sensitivity of prediction.

#### 3.2.1 DD-SIMCA

The acceptance plots of the one-class models for the COVID-19 data are presented in Figure 3. Outliers detected in the samples were removed prior to the application of the DD-SIMCA model for each preprocessed data set. The first row (Figure 3a,b) of acceptance plots corresponds to the models built with the entire NIR spectrum and shows a good performance for classifying the COVID-19 samples by using 3 PCs, while the models built with variable selection (shown in the second row, Figure 3c,d) present a better result when using 4 PCs.

**Figure 3:**
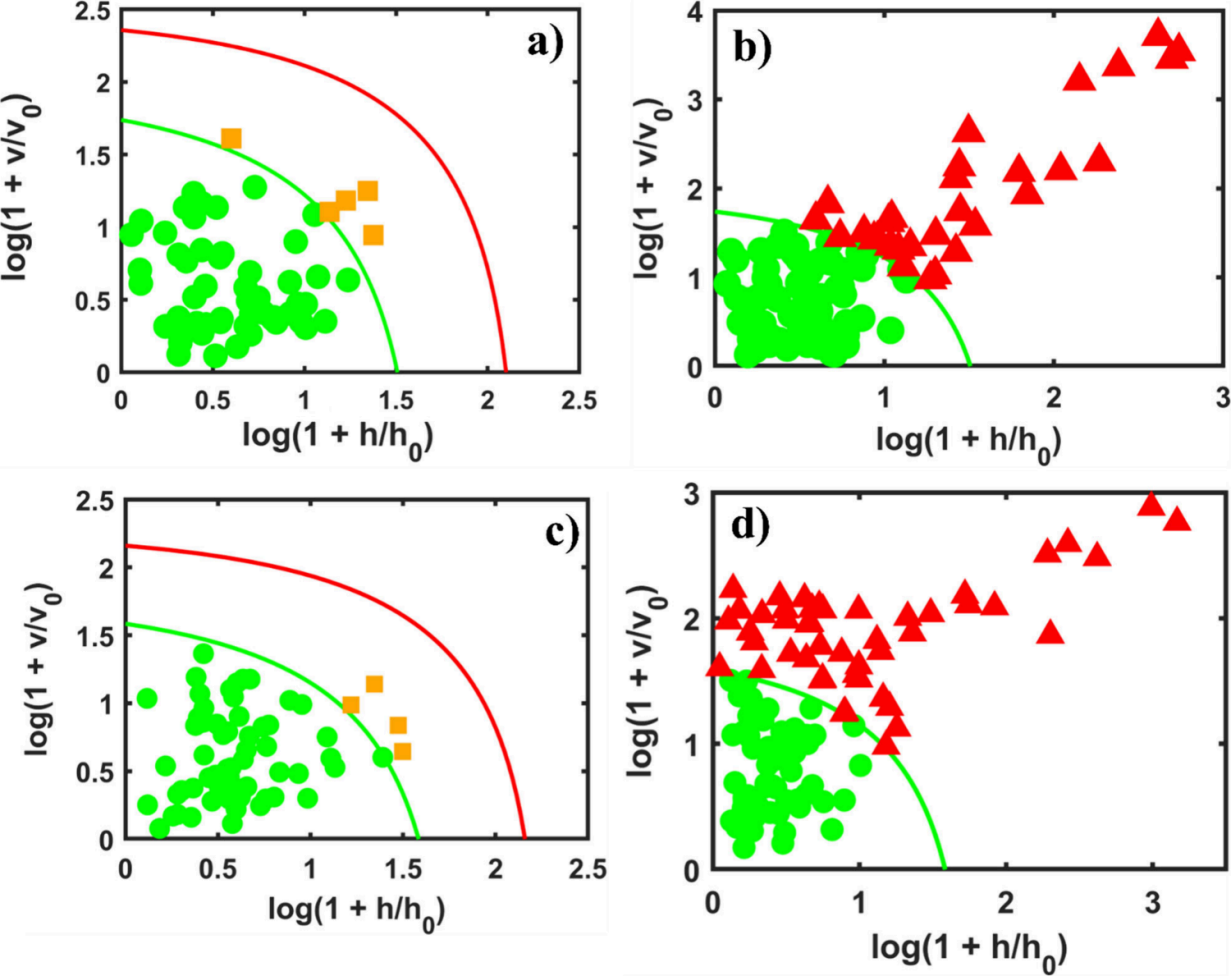
Acceptance plots of the DD-SIMCA models for the COVID-19 data. The first row shows the results without variable selection: (a) acceptance plots of the COVID-19 calibration data set and (b) COVID-19 and control test data set. This model was built using 3 PCs. The second row shows the results with variable selection: (c) acceptance plots of the COVID-19 calibration data set and (d) COVID-19 and control test data set. This model was built using 4 PCs. All models were built using the confidence level of 95% (α = 0.05).

Thus, when comparing the last acceptance plot in each row (the control data set), it can be seen that variable selection improves both the sensitivity and the selectivity for the training and test of target samples (COVID-19) and the new control test data sets. Specifically, the application of variable selection resulted in a greater number of correctly classified samples. This indicates an improved model capacity to correctly classify the control class when using the OGA variable selection method since this variable selection algorithm aims to extract useful information from large and complex data sets, such as the IR spectra, which can carry most of the important information from the samples with minimal noise, improving the classification capacity of the model.^39^

The acceptance plots for the endometriosis spectra are shown in Figure 4. Similarly to the COVID-19 samples, outliers were removed prior building the DD-SIMCA models for each preprocessed data set. The acceptance plots are shown in Figure 4. The model without variable selection used 2 PCs, while the model using the OGA used 3 PCs.

**Figure 4:**
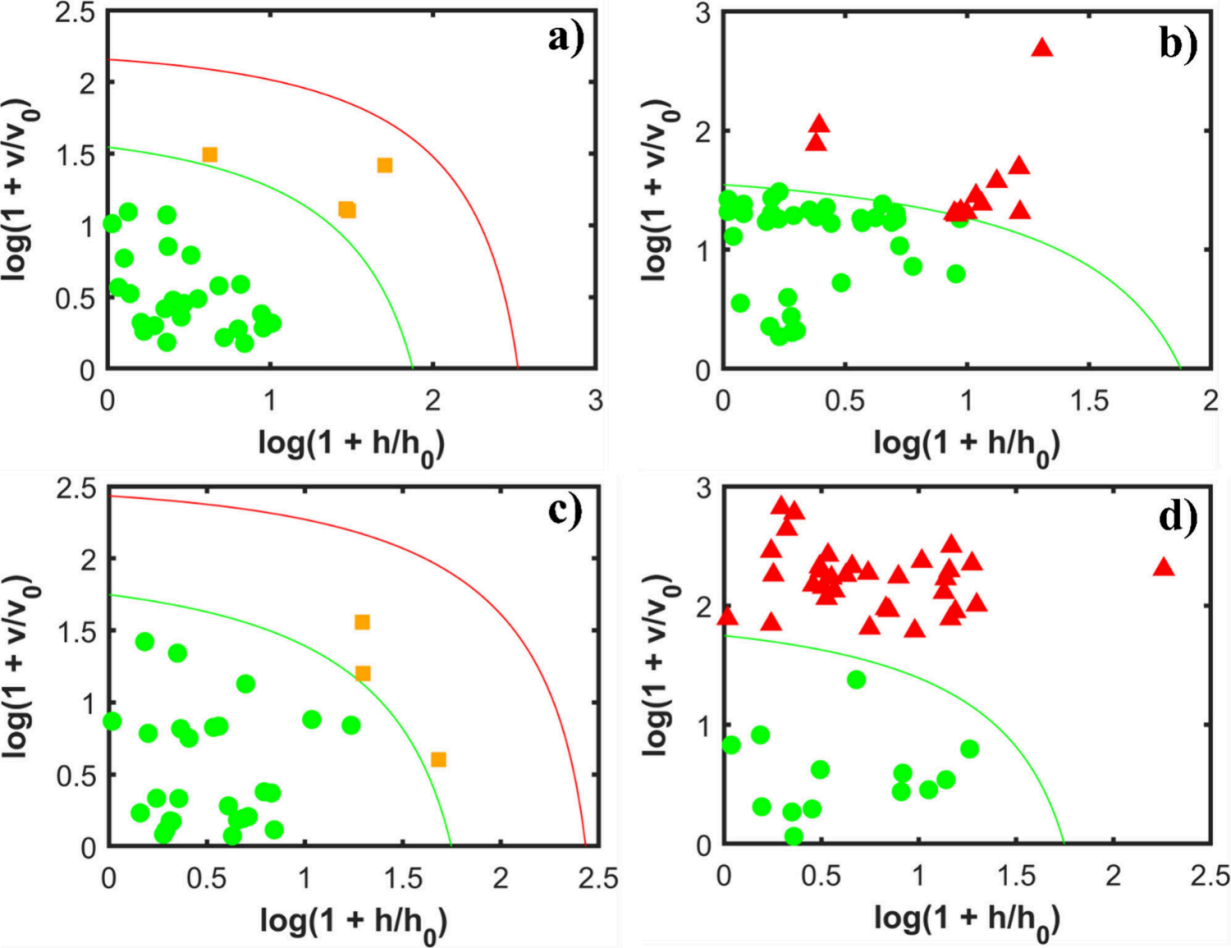
Acceptance plots of the DD-SIMCA models for the endometriosis data. The first row shows the results without variable selection: (a) acceptance plots of the endometriosis calibration data set and (b) endometriosis and control test data set. This model was built using 2 PCs. The second row shows the results with variable selection: (c) acceptance plots of the endometriosis calibration data set and (d) endometriosis and control test data set. This model was built using 3 PCs. All models were built using the confidence level of 95% (α = 0.05).

Comparing the last acceptance plot in each row, it is observed that variable selection improves both the sensitivity and selectivity levels for the training and test of the endometriosis class and the new control test data sets. The model’s ability to correctly classify the control class remains essentially unchanged when applying the variable selection.

Finally, the acceptance plots for the dengue data are presented in Figure 5. As in the other applications, the outliers were removed prior to building the DD-SIMCA models for each preprocessed data set in order to improve the classification performance of this model.^16,20^ For this data set, both models were built by using only 1 PC.

**Figure 5:**
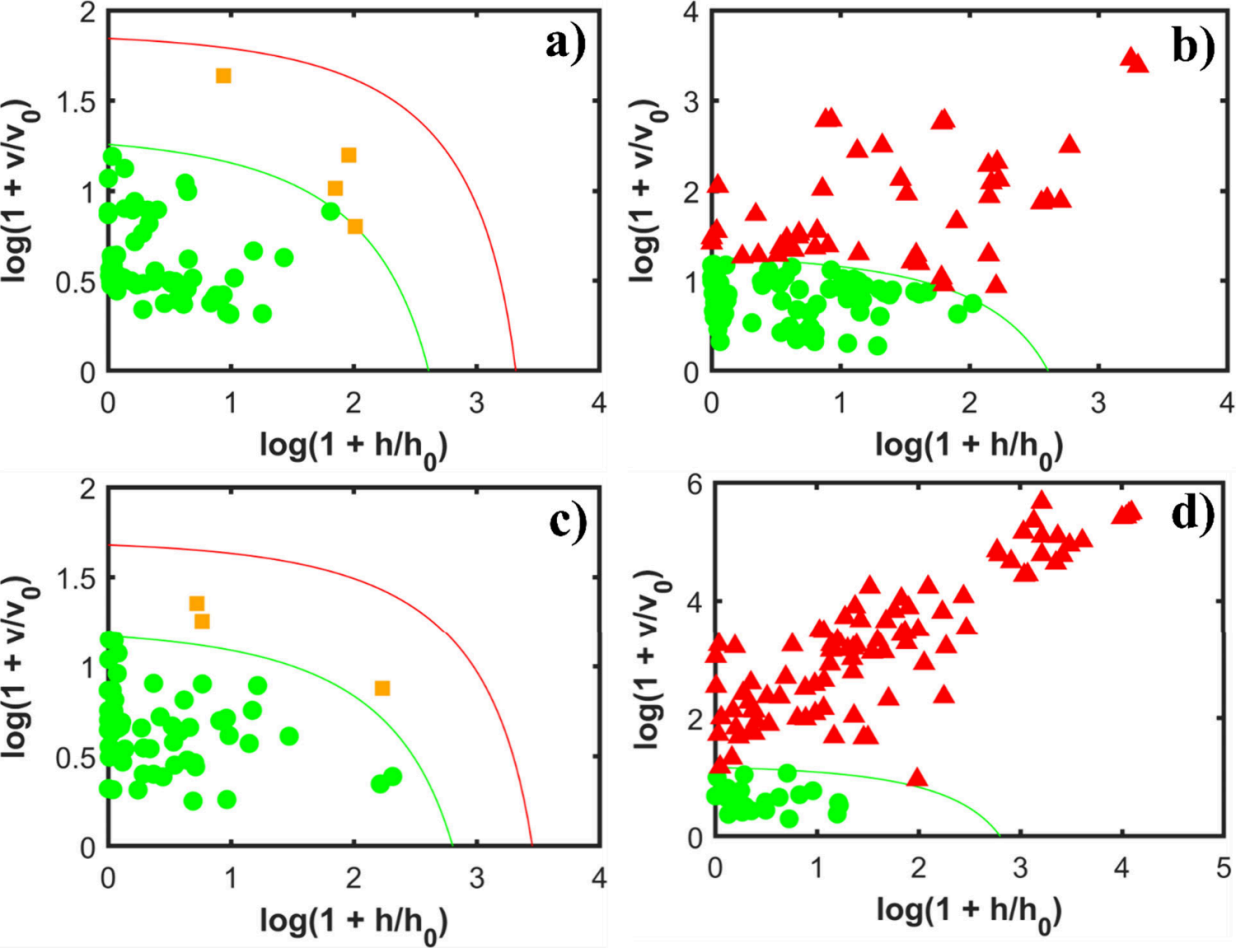
Acceptance plots of the DD-SIMCA models for the dengue data. The first row shows the results without variable selection: (a) acceptance plots of the control calibration data set and (b) control and dengue test data set. This model was built using 1 PC. The second row shows the results with variable selection: (c) acceptance plots of the control calibration data set and (d) control and dengue test data set. This model was built using 1 PC. All models were built using the confidence level of 95% (α = 0.05).

Furthermore, when comparing the last acceptance plot in each row, it is observed that variable selection improves both selectivity levels for the training and test of the control and the new dengue test data sets. The application of variable selection resulted in a greater number of correctly classified samples. This indicates an improved model capacity to correctly classify the dengue class when the OGA variable selection is used.

Although the DD-SIMCA sensitivity for COVID-19 improved following the application of OGA, the performance remains suboptimal, as only a modest enhancement in sample-classification accuracy was observed. The high sensitivity reflects that all of the COVID-19 samples were correctly identified; however, the model still produced misclassifications by erroneously assigning samples from other classes to the COVID-19 class. This same trend is also observed to a minor degree for endometriosis and could be caused by the DD-SIMCA classification threshold that prioritizes nonfalse negative classification in detriment of possible false positives. In addition, limitations associated with the spectral information for this data set based on NIR spectroscopy may contribute to the poor classification of negative samples, as NIR data have more noise and band overlapping than FTIR, for example. Thus, the inherent complexity of NIR observed in the COVID-19 and endometriosis data sets may be difficult to classify when compared to dengue (obtained by FTIR).

#### 3.2.2 PRM-OCPLS

In addition to the DD-SIMCA models, OCPLS models were also built for the three data sets studied. This model was applied in three ways, which are the ordinary OCPLS, the nonlinear Gaussian Radial Basis Function (RBF or GRBF)-OCPLS, and the OCPLS based on partial robust M-regression (PRM). However, as depicted in Table 1, the best classification performance was obtained by the PRM-OCPLS for the three IR data sets. This model probably had a better classification performance since it can detect the orthogonal and leverage outliers and then be used to develop an ordinary OCPLS model without the presence of outliers, differently from DD-SIMCA, for example.^20,22^

Figure 6 shows the plots for the PRM-OCPLS models with the COVID-19 data. As in the DD-SIMCA approach, the robust PRM models were built after removing outliers. In the first row (Figure 6a–c), the model was built using the training set without variable selection and with 5 LVs. For the second row (Figure 6d–f), the model was built using the training set with variable selection and with 7 LVs. Comparing the last plot of the first row with that from the second row, a slight improvement in the correct classification samples is observed. This slight improvement indicates that the model’s ability to correctly classify the control class remains essentially unchanged when applying the OGA, with the number of samples in region 1 (regular region) practically remaining the same.^17^

**Figure 6:**
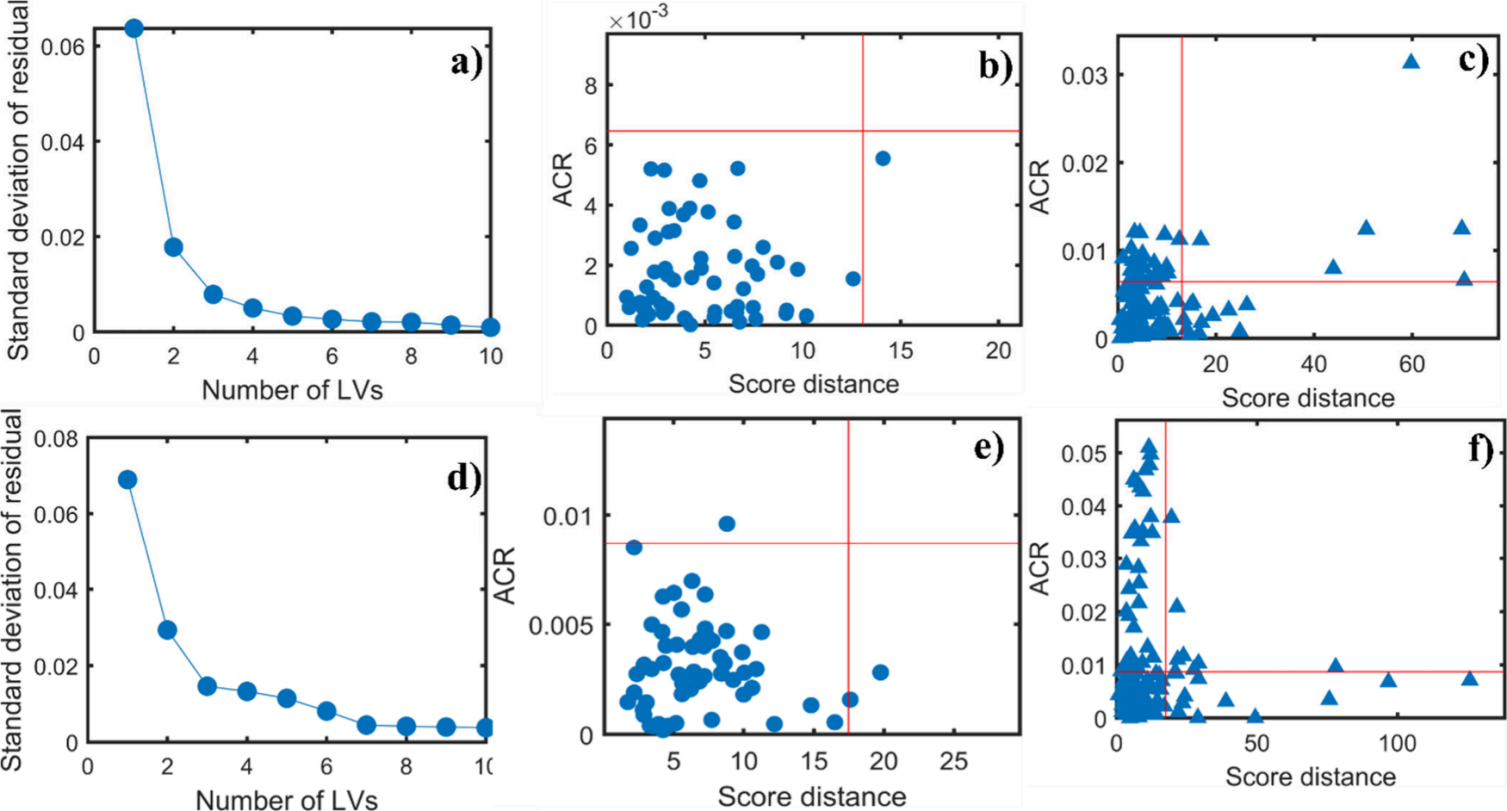
Plots of the PRM-OCPLS models based on the NIR preprocessed spectral data for COVID-19. The first row shows the results without variable selection: (a) standard deviation of residuals obtained for each LV using Traditional Cross-Validation (TCV), (b) ACR and score distances for the training data set using PRM-OCPLS with 5 LVs, and (c) ACR and score distances for the test set containing new COVID-19 samples and control data set. This model was built using 5 LVs. The second row shows the results with variable selection: (d) standard deviation of residuals obtained for each LV using Traditional Cross-Validation (TCV), (e) ACR and score distances for the training data set using PRM-OCPLS with 7 LVs, and (f) ACR and score distances for the test set containing new COVID-19 samples and control data set. This model was built using 7 LVs. All models were built using the confidence level of 95% (α = 0.05).

Figure 7 shows the plots for the PRM-OCPLS models with the endometriosis data. The robust PRM models were built after removing outliers. In the first row, the model was constructed using the training set without variable selection and with 5 LVs, while in the second row, the model was built using the training set with variable selection and 6 LVs. Comparing the last plots of both rows (Figure 7c,f), in both cases, without and with variable selection, the models were able to achieve complete separation of the control test class from the modeled target class.

**Figure 7:**
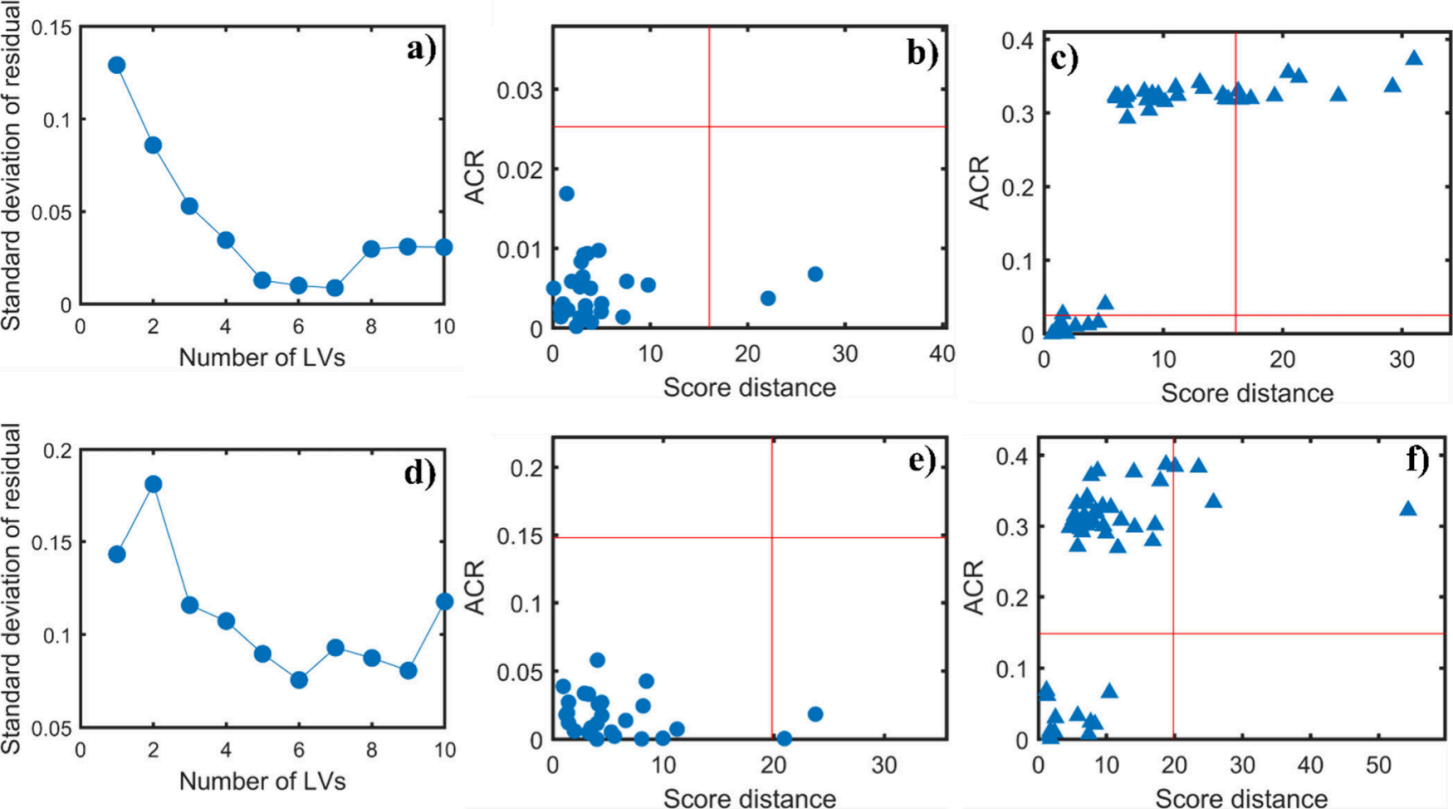
Plots of the PRM-OCPLS models based on the NIR preprocessed spectral data for endometriosis. The first row shows the results without variable selection: (a) standard deviation of residuals obtained for each LV using Traditional Cross-Validation (TCV), (b) ACR and score distances for the training data set using PRM-OCPLS with 5 LVs, and (c) ACR and score distances for the test set containing new endometriosis samples and control data set. This model was built using 5 LVs. The second row shows the results with variable selection: (d) standard deviation of residuals obtained for each LV using Traditional Cross-Validation (TCV), (e) ACR and score distances for the training data set using PRM-OCPLS with 6 LVs, and (f) ACR and score distances for the test set containing new endometriosis samples and control data set. This model was built using 6 LVs. All models were built using the confidence level of 95% (α = 0.05).

Figure 8 shows the plots for the PRM-OCPLS models of dengue data. The robust PRM models were built after removing outliers. In the first row, the model was constructed using the training set with the full spectra and with 6 LVs, while in the second row, the model was built using the training set with the variable selected by the OGA and 5 LVs. Comparing the last plots of both rows (Figure 8c,f), it is observed that this large increase in correctly classified samples indicates that the selected variables effectively discriminated dengue samples from region 1 (regular region) to region 3 (class outlier), which resulted in a better model. This better model intrinsically brings improvements provided by variable selection probably due to the extraction of important features from the data.^13,14^

**Figure 8:**
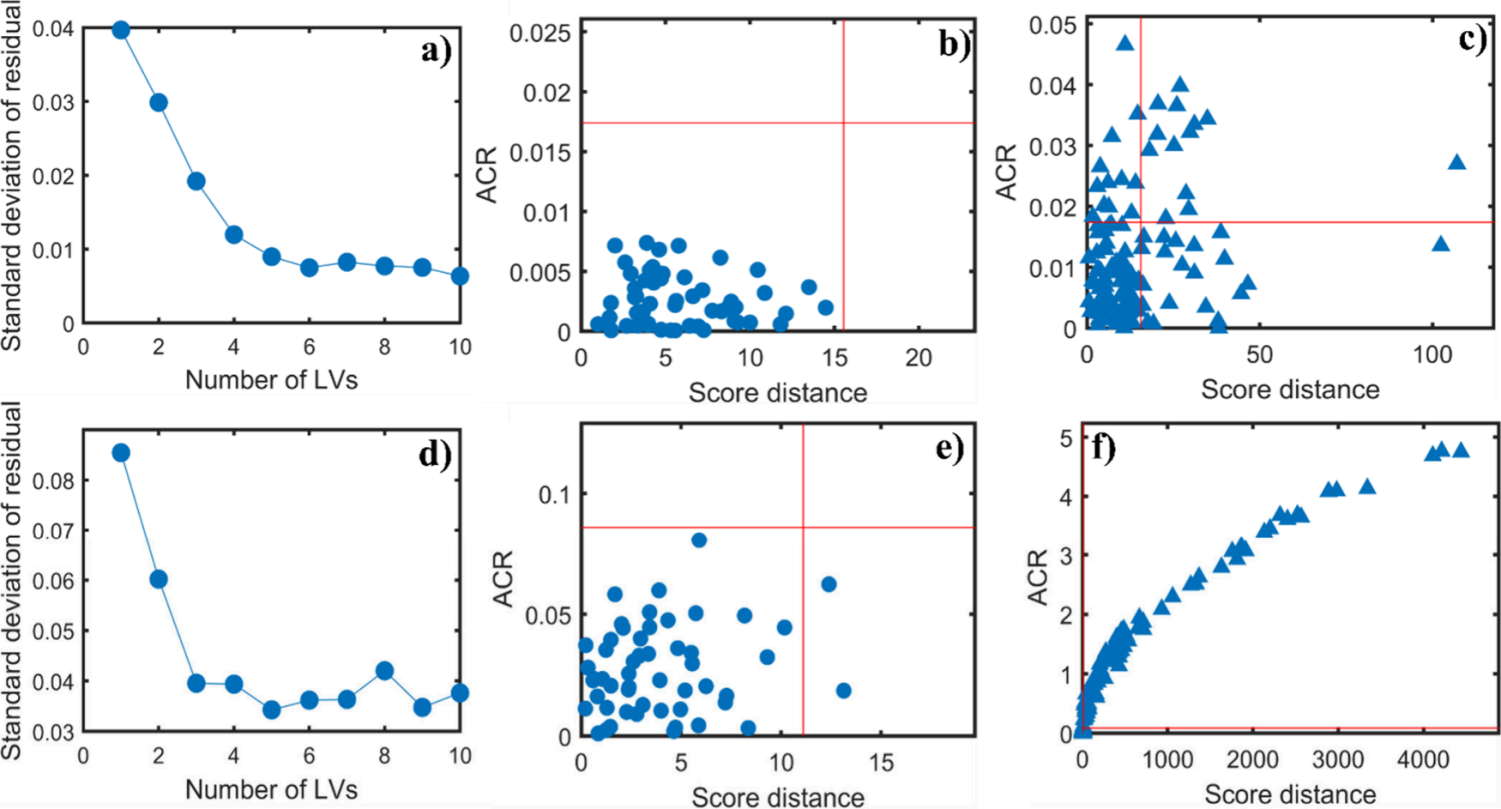
Plots of the PRM-OCPLS models based on the FTIR preprocessed spectral data for dengue. The first row shows the results without variable selection: (a) standard deviation of residuals obtained for each LV using Traditional Cross-Validation (TCV), (b) ACR and score distances for the training data set using PRM-OCPLS with 6 LVs, and (c) ACR and score distances for the test set containing new control samples and dengue data set. This model was built using 6 LVs. The second row shows the results with variable selection: (d) standard deviation of residuals obtained for each LV using Traditional Cross-Validation (TCV), (e) ACR and score distances for the training data set using PRM-OCPLS with 5 LVs, and (f) ACR and score distances for the test set containing new control samples and dengue data set. This model was built using 5 LVs. All models were built using the confidence level of 95% (α = 0.05).

### 3.3 Variables Selected by the OGA

The application of the OGA in this study is a way for improving the performance of the OCC models, as can be seen in Table 1. This happens since the variable selection algorithms can identify informative variables out of the full IR spectra, and with the removal of the irrelevant information within the system, a much simpler and parsimonious model can be obtained, without compromising its predictive ability.^40,41^ The variables selected by the OGA for the three data sets are depicted in Figure 9.

**Figure 9:**
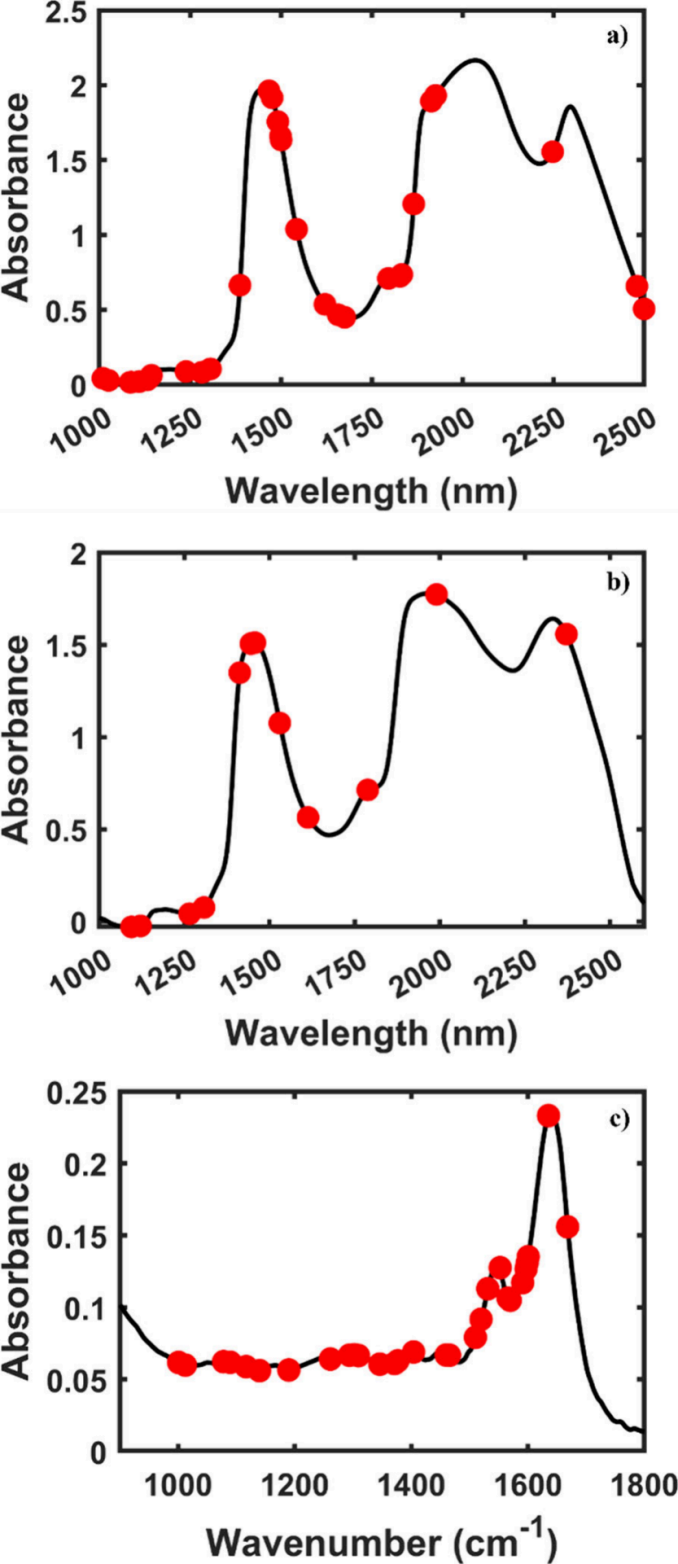
Selected variables responsible for class separation by the OGA: (a) COVID-19, (b) endometriosis, and (c) dengue.

The OGA selected 32 wavelengths that were responsible for the correct classification of the COVID-19 samples. The tentative biomarker assignments for these selected variables are shown in Table 2. Is it possible to highlight bands at 2499, 1925, 1498, and 1387 nm, which correspond to the C–H stretching and C–C and C–O–C stretching combination band of polysaccharides, −CONH– second overtone of secondary amides, −CONH_2_ first overtone of primary amides, and the C–H stretching and CH deformation from carbohydrates, respectively. These bands can be identified as potential classifier biomarkers since they correspond to some biological molecules that can be significantly altered in patients with COVID-19 due to a series of metabolic alterations in the human body by the SARS-CoV-2 virus.^42–46^

**Table 2:** Wavelengths Selected by OGA Applied to the Preprocessed NIR Data for Differentiating Healthy Controls vs COVID-19 Samples

Selected Wavelength (nm)	Tentative Assignment
2499	C–H stretching and C–C and C–O–C stretching combination - polysaccharides
2480	C–H (3 deformation CH_3_) - C–H methyl C–H, associated with aromatic (ArCH_3_) Hydrocarbons, aromatic
2248	OH stretching and OH deformation
1925	–CONH– secondary amides - second overtone of amide
1914	COOH carboxylic acids - 2× C=O stret. (carboxylic acids)
1865	C–Cl stretch, chlorinated hydrocarbons
1832	CH– stretch first overtone of carbonyl compounds
1827	O–H/C–H combination - O–H stretching and C–O stretching
1795	–CH_2_ methylene combination - first overtone of sym. stret.
1675	C=C first overtone vinyl group
1657	C=C first overtone vinyl group
1621	C–H stretch first overtone of terminal methylene groups of vinyl
1543	Bound - OH alcohol - first overtone intramolecular hydrogen bond
1500	–NH_2_ primary amines - first overtone of NH_2_ antisym. stret.
1498	–CONH_2_ primary amides - first overtone of NH_2_ sym. stret.
1491	–NH secondary amines - first overtone
1475	Bound - OH alcohol - first overtone - intermolecular hydrogen bond
1467	–NH_2_ primary amines - first overtone ArNH_2_
1387	2 C–H stretching and CH deformation - carbohydrates
1306	NH and NH_2_ stretching mode - first overtone and combination band
1284	OH first overtone - albumin
1282	OH first overtone - albumin
1238	C–H stretching overtone
1143	C=C alkenes - second overtone - vinyl group
1136	–C=CH alhynes (ethynyle) second overtone
1131	CH (aromatic) second overtone
1110	C=C second overtone vinyl group
1109	C–H stretch second overtone of the therminal methylene in allyl stearate
1087	–CONH– secondary amides - second overtone - hydrogen bond
1085	CH (aromatic) combination band
1025	–CONH_2_– primary amides - second overtone - intermolecular hydrogen bond
1009	–CONH_2_– primary amides - second overtone of NH_2_ symmetric stretching

When applying the algorithm to the endometriosis data set, the OGA selected 15 wavelengths that were responsible for the correct classification of the endometriosis samples. The tentative biomarker assignments for these selected variables are shown in Table 3. The band selected at 2371 nm corresponds to CH stretching and C–C stretching combination from lipids, and the band at 1990 nm can be associated with the combination band of NH stretching + amide. The bands at 1612 and 1095 nm are related to the first overtone of the intermolecular hydrogen bond from primary amines and the second overtone of the hydrogen bond for secondary amides, respectively. These variables are in accordance with previous results obtained by the GA for discriminant analysis.^30^

**Table 3:** Wavelengths Selected by OGA Applied to the NIR Data to Discriminate between Healthy Controls and Endometriosis Samples

Selected Wavelength (nm)	Tentative Assignment
2371	CH stretching and C–C stretching combination from lipids
1990	Combination band of NH stretching + amide
1788	First overtone of –CH_3_ methyl
1612	First overtone of intermolecular hydrogen bond from primary amines
1529	First overtone of intermolecular hydrogen bond from primary amines
1456	First overtone of NH_2_ antisymmetric stretching
1448	First overtone of NH_2_ antisymmetric stretching
1445	First overtone of NH_2_ antisymmetric stretching
1412	Combination band of –CH_2_ methylene (2× CH stretching + CH bending)
1307	NH and NH_2_ stretching mode - first overtone and combination band
1265	NH first overtone
1210	Second overtone of –CH_2_ methylene
1095	Second overtone of the hydrogen bond for secondary amides
995	Second overtone of –NH_2_ primary amines (ArNH_2_)
944	Second overtone of OH stretching

Finally, 31 wavenumbers were selected with the OGA for the dengue data set, which were responsible for the correct classification of the virus samples. The tentative biomarker assignments for these selected variables are listed in Table 4. By analyzing the selected wavenumbers, it is possible to conclude that OGA attributed the main variables for one-class classification between the classes to phosphodiester groups of nucleic acids (phosphate I in RNA) at ∼1078 cm^–1^; amide I (∼1668 cm^–1^); and amide II (∼1552 cm^–1^), which are in accordance with previous studies involving variable selection for virus discrimination.^36^

**Table 4:** Wavenumbers Selected by OGA Applied to the Preprocessed ATR-FTIR Data for Differentiating Healthy Controls vs Dengue Samples

Selected Wavenumber (cm^–1^)	Tentative Assignment
1668	Amide I (antiparallel β-sheet)
1635	β-sheet structure of amide I
1600	C=O stretching (lipids)
1598	C=N, NH_2_ adenine
1596	Methylated nucleotides
1591	C=N, NH_2_ adenine
1569	Amide II
1566	Ring base
1552	Amide II
1531	Stretching C=N, C=C
1519	Amide II
1510	In-plane CH bending vibration from the phenyl rings
1465	CH_2_ scissoring mode of the acyl chain of lipid
1459	Asymmetric CH_3_ bending modes of the methyl groups of proteins
1404	CH_3_ asymmetric deformation
1377	Stretching C–O, deformation C–H, deformation N–H
1373	Stretching C–N cytosine, guanine
1371	Stretching C–O, deformation C–H, deformation N–H
1346	CH_2_ wagging
1309	Amide III
1303	Amide III
1301	Deformation N–H cytosine
1294	Deformation N–H cytosine
1261	PO_2_^–^ asymmetric (phosphate I)
1189	Amide III
1139	Oligosaccharide C–OH stretching band
1116	C–O stretching vibration of C–OH group of ribose (RNA)
1089	Stretching PO_2_^–^ symmetric in RNA
1078	Phosphate I in RNA
1012	CH_α,α′_ out-of-plane bending and C_α_=C_α′_ torsion
1000	Protein amide I absorption

## 4 Conclusions

In this study, one-class classifier models associated with a new variable selection strategy, the one-class genetic algorithm (OGA), were applied to clinical data. Although both models provided similar classification results, for the COVID-19 data, the OGA-PRM-OCPLS showed more robust classification than DD-SIMCA. The same happened for the endometriosis and dengue data. Furthermore, the wavelength and wavenumbers selected in the three data sets studied could be related to biomarkers capable of differentiating the classes, providing valuable biochemical information for the detection of COVID-19, endometriosis, and dengue. Herein, for the first time, the OGA associated with the OCC models is reported, such as DD-SIMCA and OCPLS, and they can be implemented as screening models in clinical applications using spectroscopy, highlighting their potential for less expensive and less time-consuming analyses. However, some limitations including the small sample size, need for independent data set validation, and nondeterministic nature of the OGA must be taken into account before considering this approach for generalizability and clinical implementation.

## Supplementary Material



## Notes

Additional results, including OCPLS and GRBF-OCPLS model results, DD-SIMCA extreme plots for determining the optimal number of PCs, and DD-SIMCA auto models and extreme plots (PDF)
