# Supplementary material for: One-Class Genetic Algorithm for Authentication Analysis of Spectrochemical Data
Source: ACS Omega. 2025 Dec 31;11(2):2628–40. doi: 10.1021/acsomega.5c07696 (PMC12824936; doi:10.1021/acsomega.5c07696)
Supplement: Supplementary file 1 [file ao5c07696_si_001.pdf]

One-class genetic algorithm for authentication analysis of spectrochemical data

José R. de Morais Filho[1], Camilo de L. M. de Morais[2], Anne B. F. Câmara[1], Kássio M. G. de Lima[1,*]

[1]Biological Chemistry and Chemometrics, Institute of Chemistry, Federal University of Rio Grande do Norte, Natal, RN, 5072-970, Brazil.

[2]Center for Education, Science and Technology of the Inhamuns Region, State University of Ceará, Tauá, CE, 63660-000, Brazil.

*Corresponding author: Email: kassio.lima@ufrn.br

OCPLS

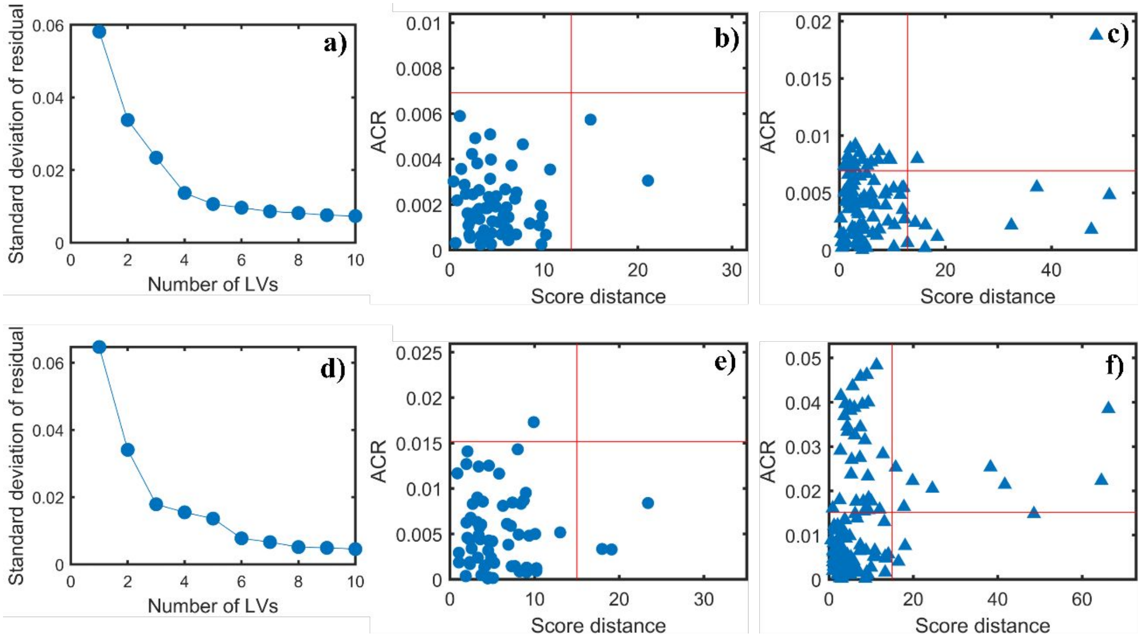

Figure S1. Plots of the OCPLS models based on the NIR preprocessed spectral data. The first row shows the results without variable selection: (a) Standard deviation of residuals obtained for each LV using Traditional Cross-Validation (TCV), (b) ACR and score distances for the training dataset using OCPLS with 5 LVs, (c) ACR and score distances for the test set containing new COVID-19 samples and Control dataset. This model was built using 5 LVs. The second row shows the results with variable selection: (d) Standard deviation of residuals obtained for each LV using Traditional Cross-Validation (TCV), (e) ACR and score distances for the training dataset using OCPLS with 6 LVs, (f) ACR and score distances for the test set containing new COVID-19 samples and Control dataset. This model was built using 6 LVs. All models were built using the confidence level of 95 % (α = 0.05).

GRBF-OCPLS

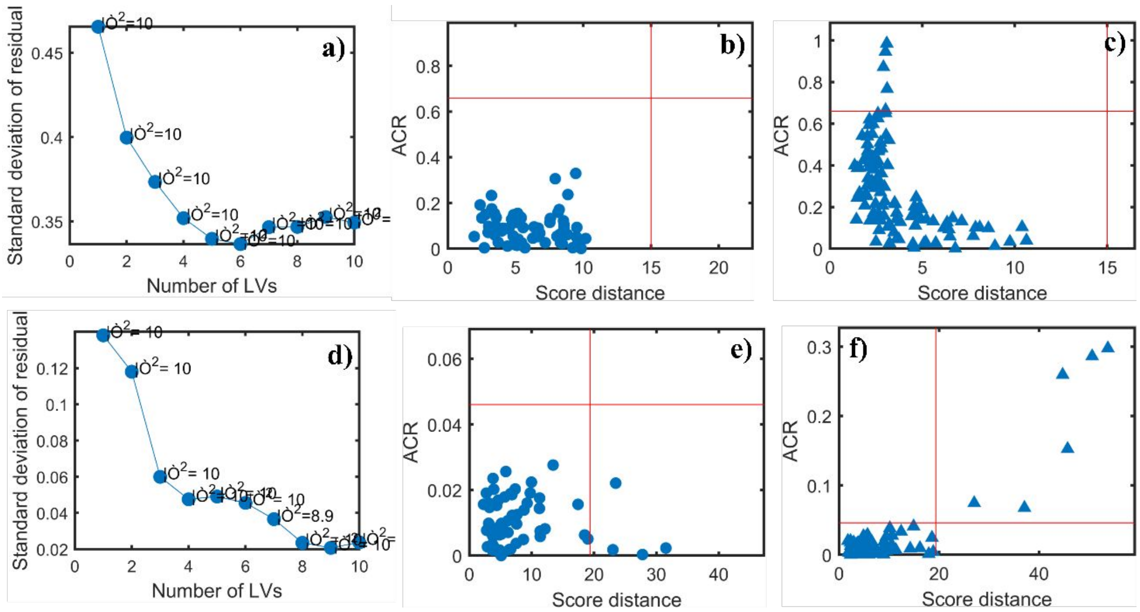

Figure S2. Plots of the GRBF-OCPLS models based on the NIR preprocessed spectral data. The first row shows the results without variable selection: (a) Standard deviation of residuals obtained for each LV using Traditional Cross-Validation (TCV), (b) ACR and score distances for the training dataset using GRBF-OCPLS with 6 LVs, (c) ACR and score distances for the test set containing new COVID-19 samples and Control dataset. This model was built using 6 LVs. The second row shows the results with variable selection: (d) Standard deviation of residuals obtained for each LV using Traditional Cross-Validation (TCV), (e) ACR and score distances for the training dataset using GRBF-OCPLS with 8 LVs, (f) ACR and score distances for the test set containing new COVID-19 samples and Control dataset. This model was built using 8 LVs. All models were built using the confidence level of 95 % (α = 0.05).

Extreme Plot DD-SIMCA

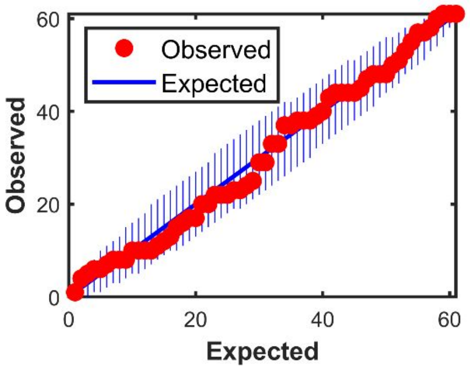

Figure S3. Extreme Plot of the Training set COVID-19.

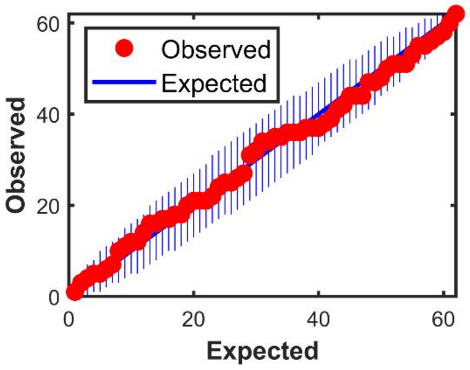

Figure S4. Extreme Plot of the Training-OGA set COVID-19.

Acceptance Plot COVID-19 DD-SIMCA Auto.

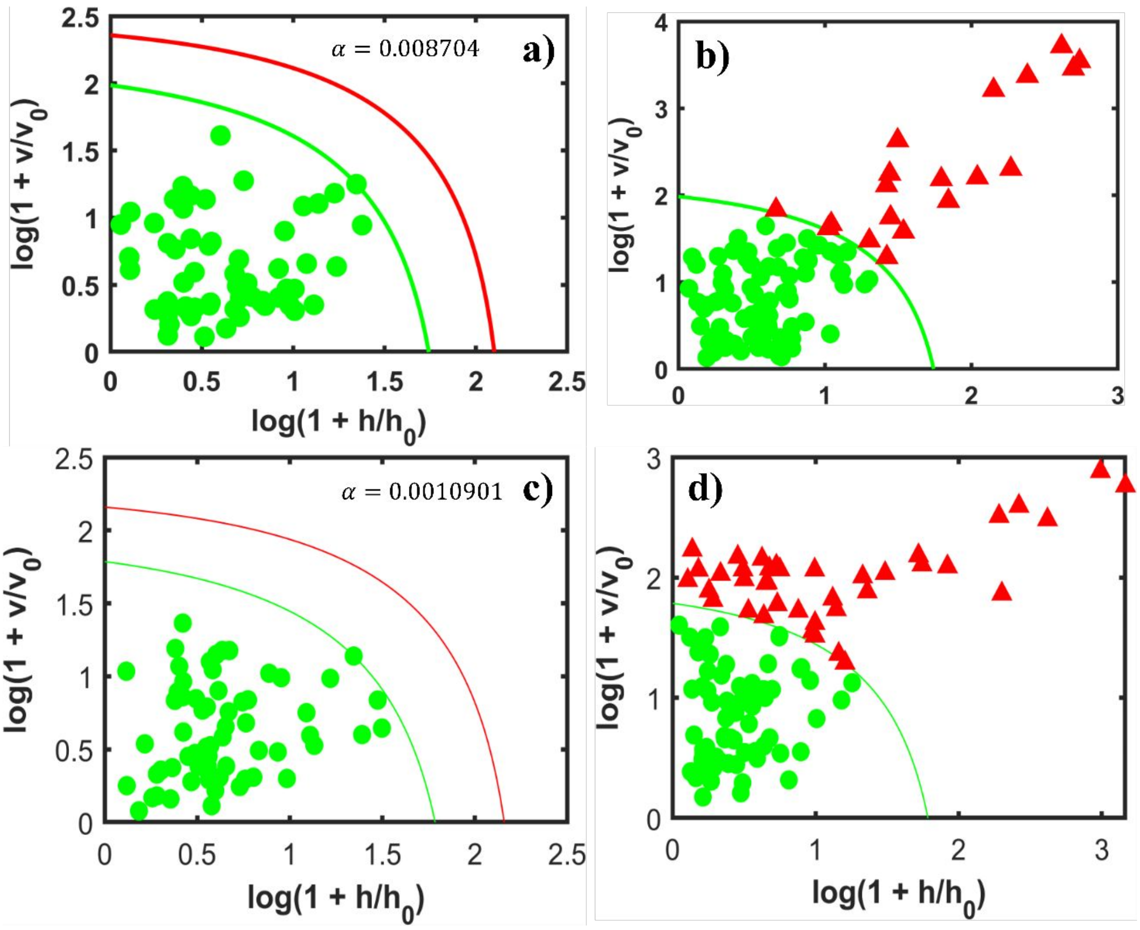

Figure S5. Acceptance plot DD-SIMCA Auto models. The first row shows the results without variable selection: (a) acceptance plot of the COVID-19 calibration dataset, (b) COVID-19 and Control Test dataset. Confidence level α = 0.008704. This model was built using 3 PCs. The second row shows the results with variable selection: (c) acceptance plot of the COVID-19 calibration dataset, (d) COVID-19 and Control Test dataset. Confidence level α = 0.0010901. This model was built using 4 PCs.

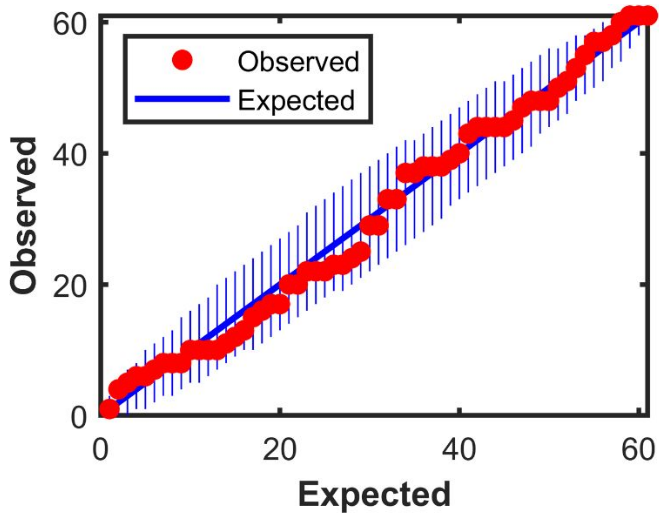

Figure S6. Extreme Plot DD-SIMCA Auto of the Training set COVID-19.

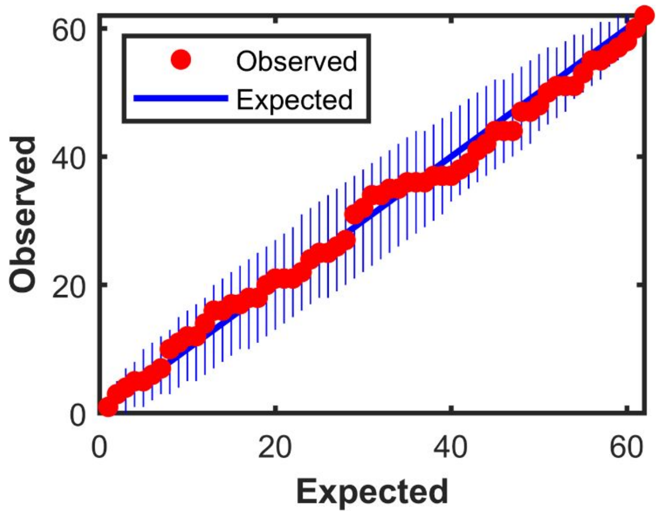

Figure S7. Extreme Plot DD-SIMCA Auto of the Training-OGA set COVID-19.

Endometriosis

OCPLS

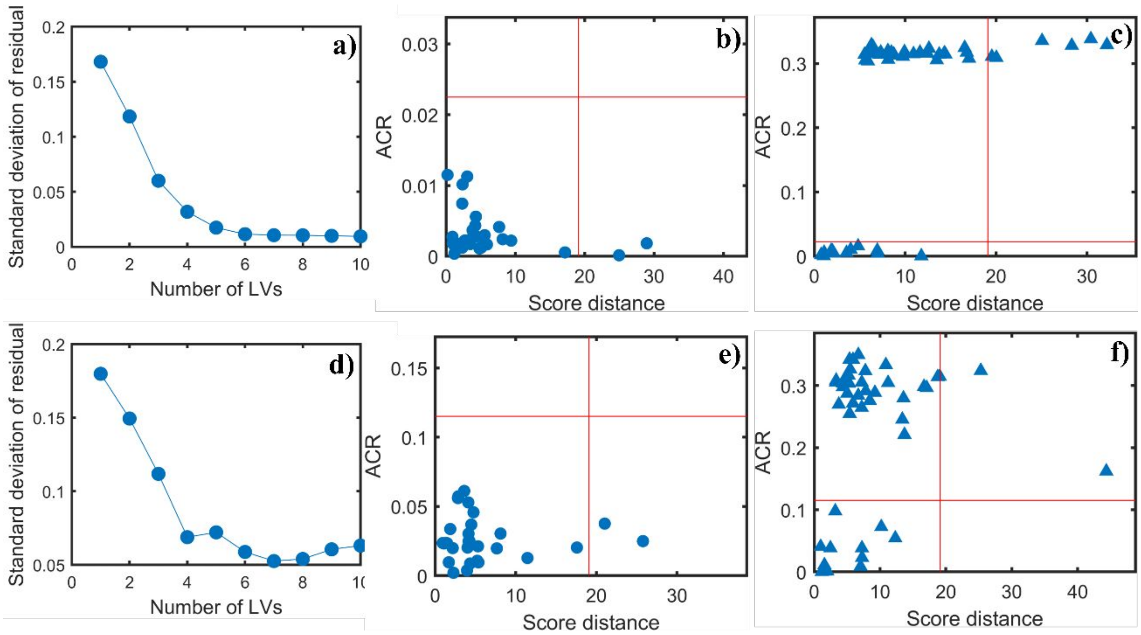

Figure S8: Plots of the OCPLS models based on the NIR preprocessed spectral data. The first row shows the results without variable selection: (a) Standard deviation of residuals obtained for each LV using Traditional Cross-Validation (TCV), (b) ACR and score distances for the training dataset using OCPLS with 6 LVs, (c) ACR and score distances for the test set containing new Endometriosis samples and Control dataset. This model was built using 6 LVs. The second row shows the results with variable selection: (d) Standard deviation of residuals obtained for each LV using Traditional Cross-Validation (TCV), (e) ACR and score distances for the training dataset using OCPLS with 6 LVs, (f) ACR and score distances for the test set containing new Endometriosis samples and Control dataset. This model was built using 6 LVs. All models were built using the confidence level of 95 % (α = 0.05).

GRBF-OCPLS

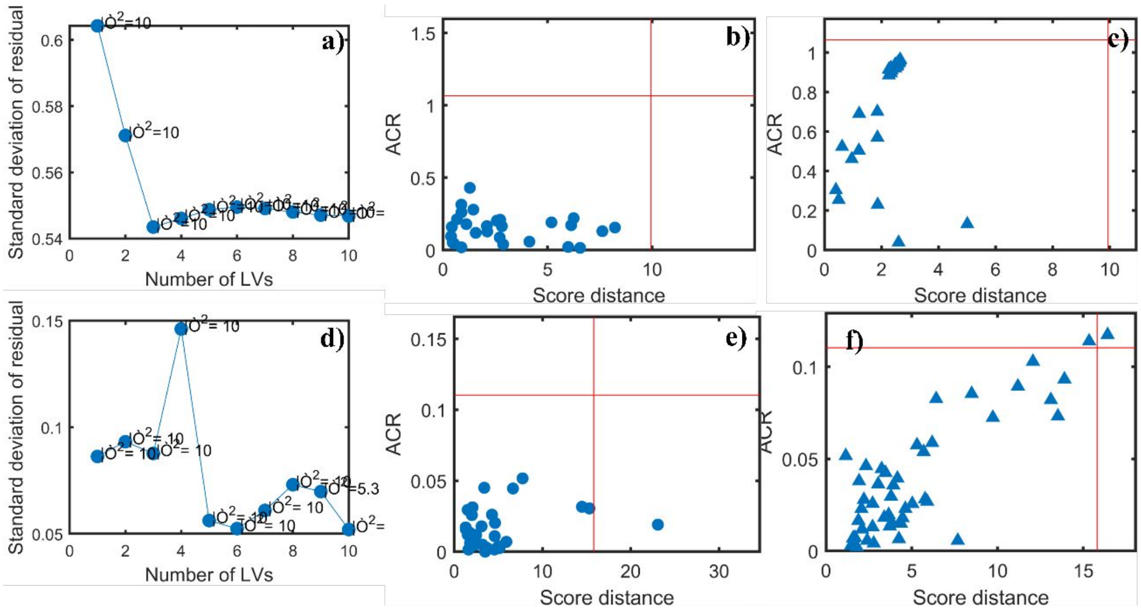

Figure S9: Plots of the GRBF-OCPLS models based on the NIR preprocessed spectral data. The first row shows the results without variable selection: (a) Standard deviation of residuals obtained for each LV using Traditional Cross-Validation (TCV), (b) ACR and score distances for the training dataset using GRBF-OCPLS with 3 LVs, (c) ACR and score distances for the test set containing new Endometriosis samples and Control dataset. This model was built using 3 LVs. The second row shows the results with

variable selection: (d) Standard deviation of residuals obtained for each LV using Traditional Cross-Validation (TCV), (e) ACR and score distances for the training dataset using GRBF-OCPLS with 5 LVs, (f) ACR and score distances for the test set containing new Endometriosis samples and Control dataset. This model was built using 5 LVs. All models were built using the confidence level of 95 % (α = 0.05).

Extreme Plot DD-SIMCA

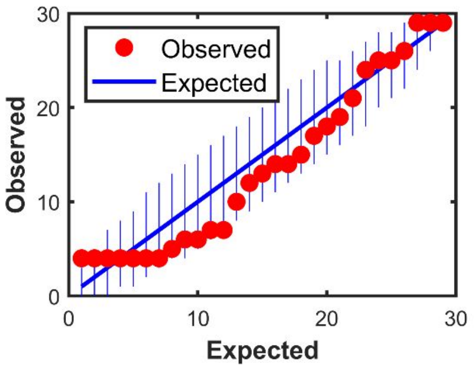

Figure S10. Extreme Plot of the Training set Endometriosis.

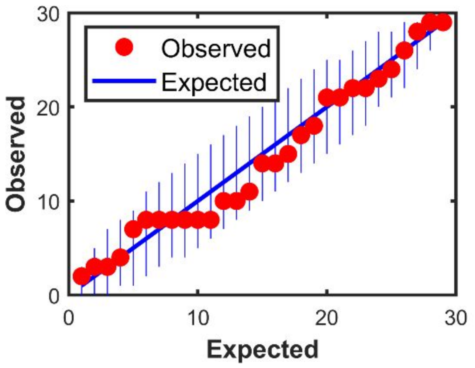

Figure S11. Extreme Plot of the Training-OGA set Endometriosis.

Acceptance Plot Endometriosis DD-SIMCA Auto.

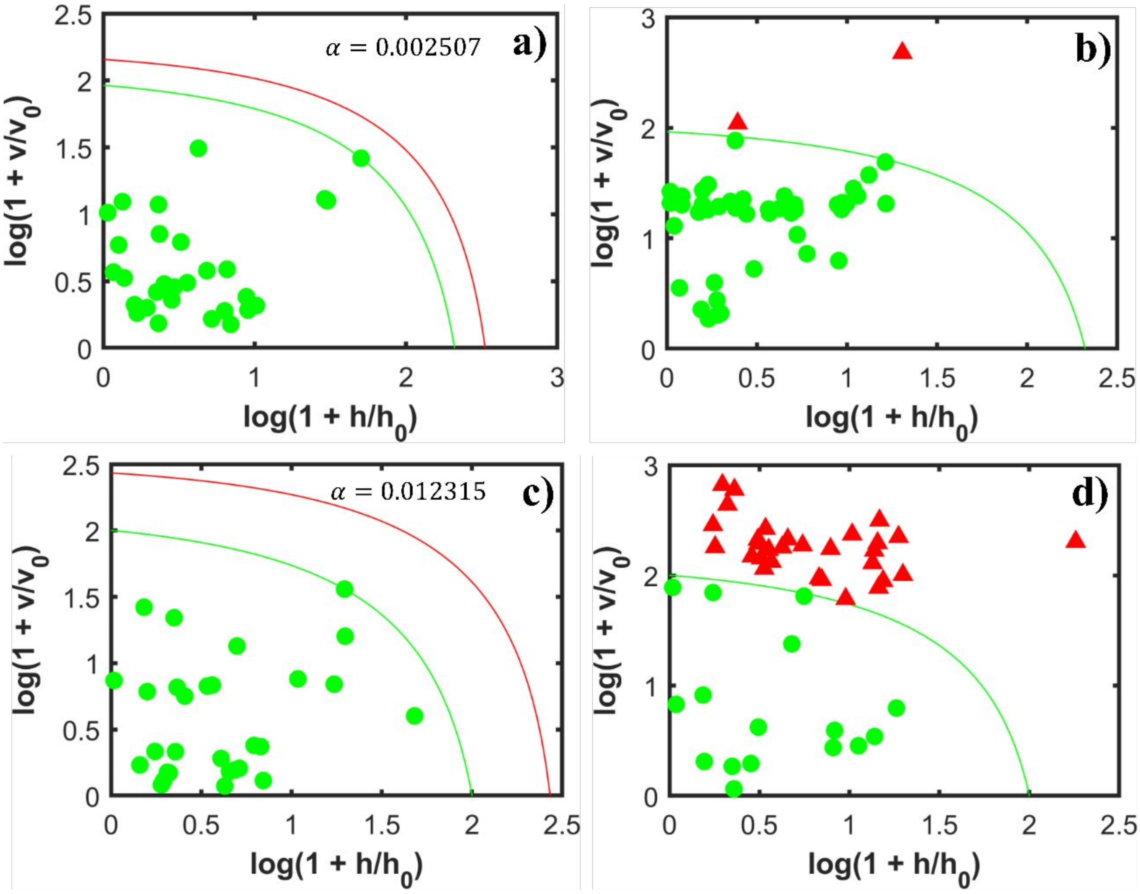

Figure S12. Acceptance plot DD-SIMCA Auto models. The first row shows the results without variable selection: (a) acceptance plot of the Endometriosis calibration dataset, (b) Endometriosis and Control Test dataset. Confidence level α = 0.002507. This model was built using 2 PCs. The second row shows the results with variable selection: (c) acceptance plot of the Endometriosis calibration dataset, (d) Endometriosis and Control Test dataset. Confidence level α = 0.012315. This model was built using 3 PCs.

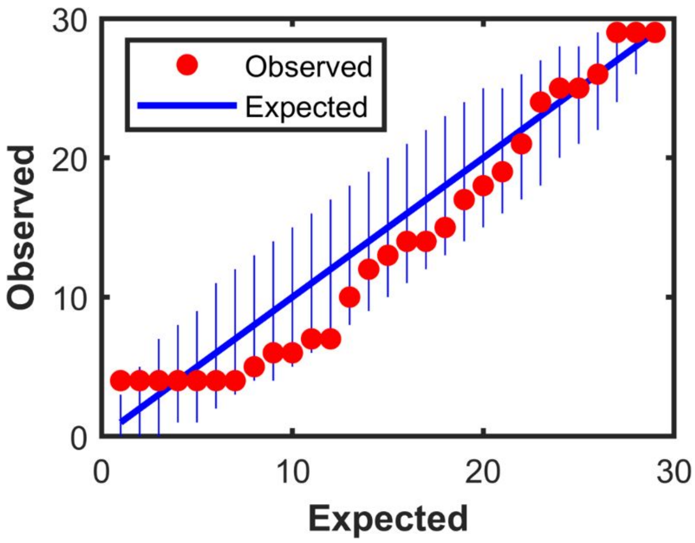

Figure S13. Extreme Plot DD-SIMCA Auto of the Training set Endometriosis.

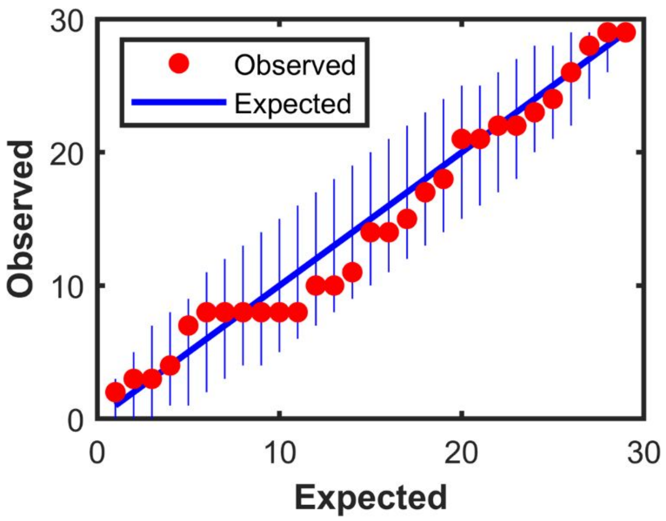

Figure S14. Extreme Plot DD-SIMCA Auto of the Training-OGA set Endometriosis.

Dengue

OCPLS

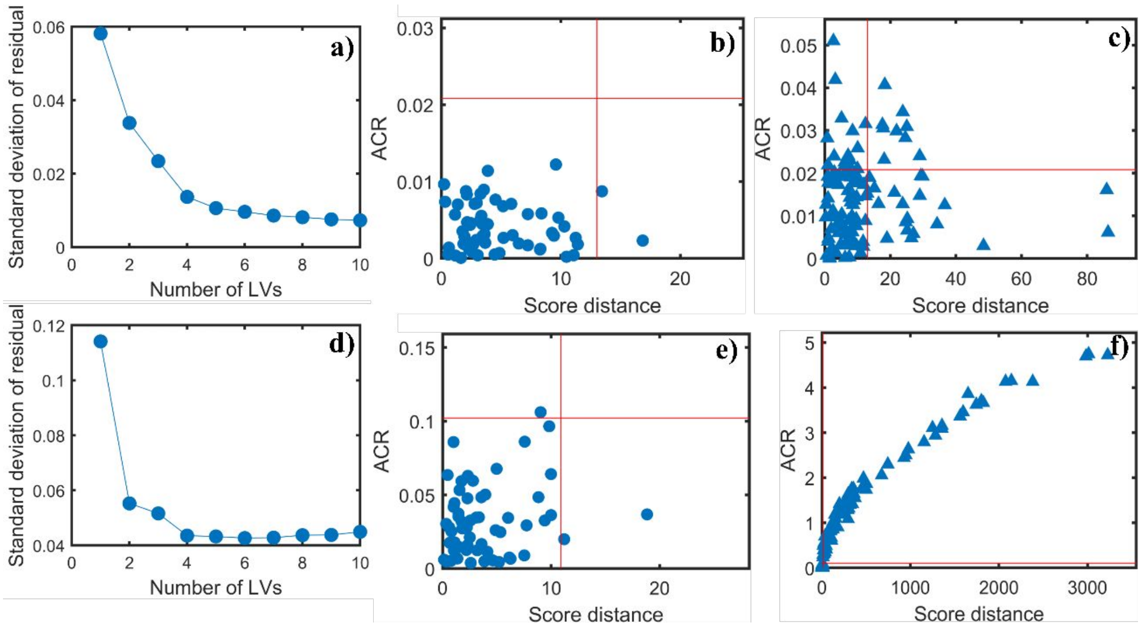

Figure S15. Plots of the OCPLS models based on the NIR preprocessed spectral data. The first row shows the results without variable selection: (a) Standard deviation of residuals obtained for each LV using Traditional Cross-Validation (TCV), (b) ACR and score distances for the training dataset using OCPLS with 5 LVs, (c) ACR and score distances for the test set containing new Control samples and Dengue dataset. This model was built using 5 LVs. The second row shows the results with variable selection: (d) Standard deviation of residuals obtained for each LV using Traditional Cross-Validation (TCV), (e) ACR and score distances for the training dataset using OCPLS with 4 LVs, (f) ACR and score distances for the test set containing new Control samples and Dengue dataset. This model was built using 4 LVs. All models were built using the confidence level of 95 % (α = 0.05).

GRBF-OCPLS

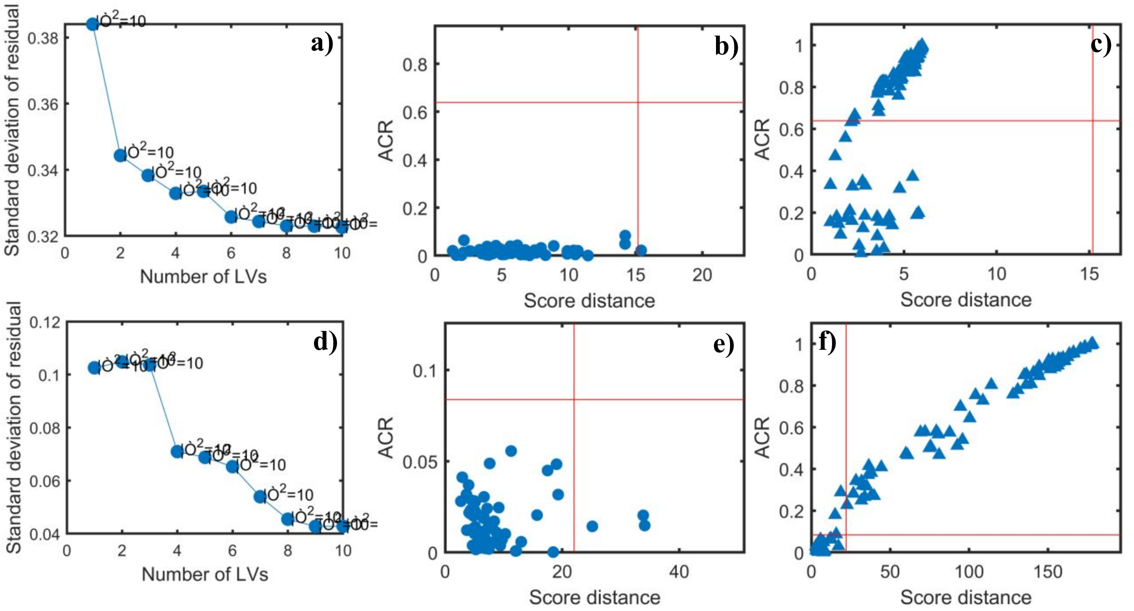

Figure S16. Plots of the GRBF-OCPLS models based on the NIR preprocessed spectral data. The first row shows the results without variable selection: (a) Standard deviation of residuals obtained for each LV using Traditional Cross-Validation (TCV), (b) ACR and score distances for the training dataset using GRBF-OCPLS with 6 LVs, (c) ACR and score distances for the test set containing new Control samples and Dengue dataset. This model was built using 6 LVs. The second row shows the results with variable selection: (d) Standard deviation of residuals obtained for each LV using Traditional Cross-Validation (TCV), (e) ACR and score distances for the training dataset using GRBF-OCPLS with 9 LVs, (f) ACR and score distances for the test set containing new Control samples and Dengue dataset. This model was built using 9 LVs. All models were built using the confidence level of 95 % ($\alpha = 0.05$).

Extreme Plot DD-SIMCA

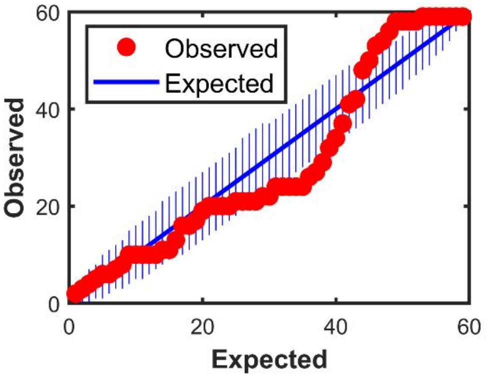

Figure S17. Extreme Plot of the Training set Dengue.

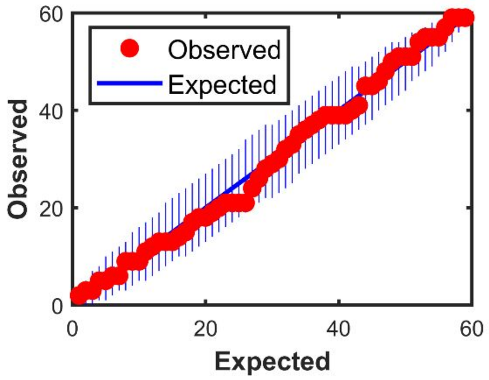

Figure S18. Extreme Plot of the Training-OGA set Dengue.

Acceptance Plot Dengue DD-SIMCA Auto.

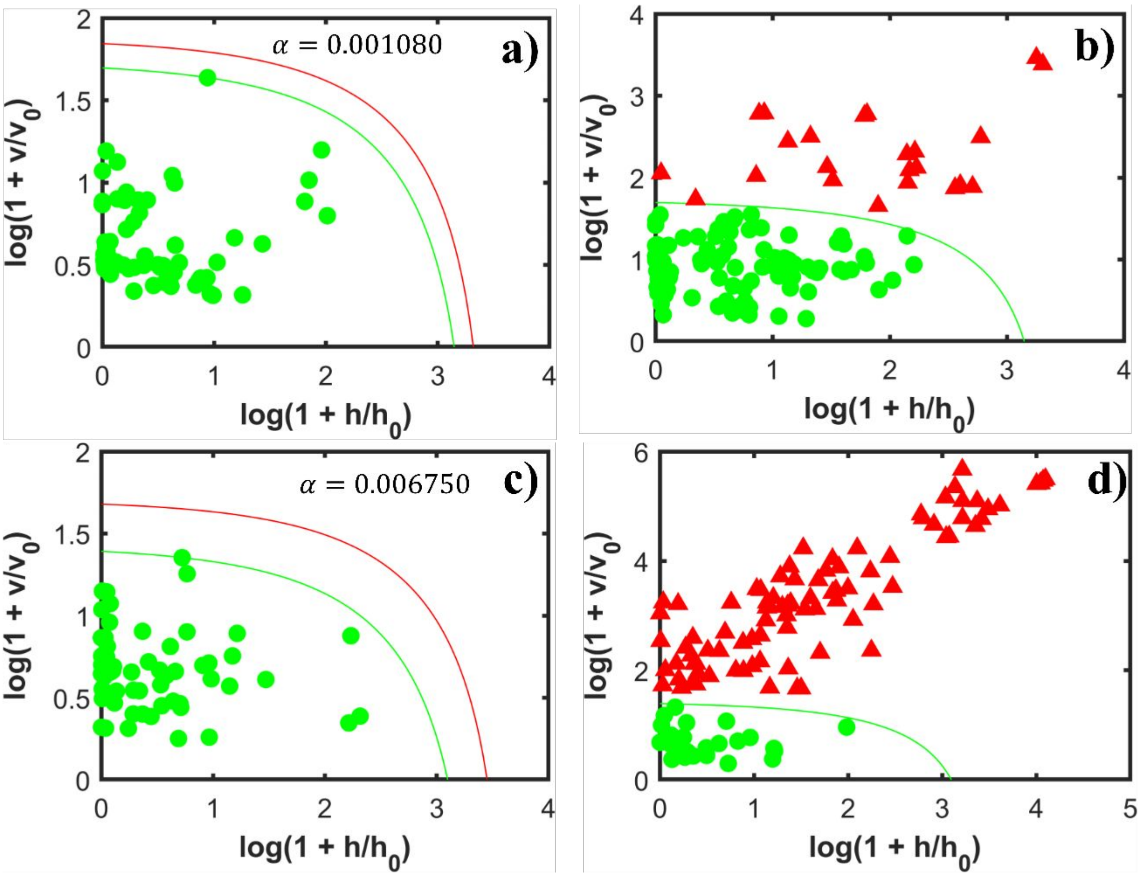

Figure S19. Acceptance plot of the DD-SIMCA Auto models. The first row shows the results without variable selection: (a) acceptance plot of the Control calibration dataset, (b) Control and Dengue Test dataset. Confidence level α = 0.001080. This model was built using 1 PC. The second row shows the results with variable selection: (c) acceptance plot of the Control calibration dataset, (d) Control and Dengue Test dataset. Confidence level α = 0.006750. This model was built using 1 PC.

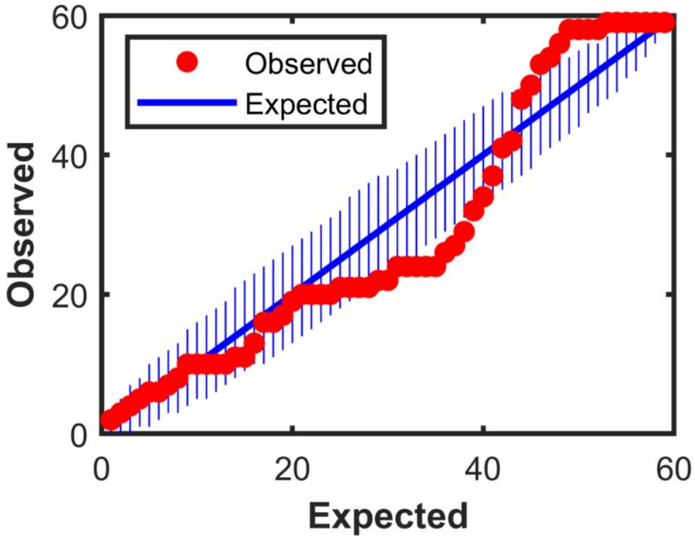

Figure S20. Extreme Plot DD-SIMCA Auto of the Training set Dengue.

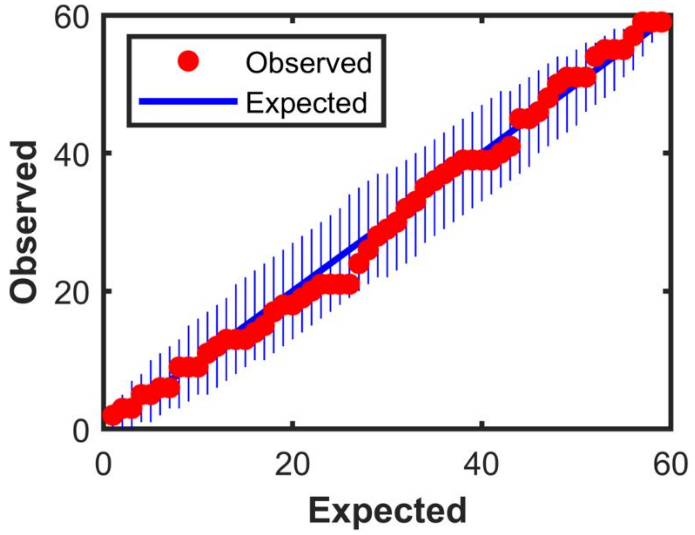

Figure S21. Extreme Plot DD-SIMCA Auto of the Training-OGA set Dengue.
